# Characterization of Microbial Dysbiosis and Metabolomic Changes in Dogs with Acute Diarrhea

**DOI:** 10.1371/journal.pone.0127259

**Published:** 2015-05-22

**Authors:** Blake C. Guard, James W. Barr, Lavanya Reddivari, Cory Klemashevich, Arul Jayaraman, Jörg M. Steiner, Jairam Vanamala, Jan S. Suchodolski

**Affiliations:** 1 Gastrointestinal Laboratory, Department of Small Animal Clinical Sciences, College of Veterinary Medicine and Biomedical Sciences, Texas A&M University, College Station, Texas, United States of America; 2 Emergency and Critical Care, Department of Small Animal Clinical Sciences, College of Veterinary Medicine and Biomedical Sciences, Texas A&M University, College Station, Texas, United States of America; 3 Department of Plant Science, Penn State University, University Park, Pennsylvania, United States of America; 4 Artie McFerrin Department of Chemical Engineering, Texas A&M University, College Station, Texas, United States of America; 5 Department of Microbial Pathogenesis and Immunology, Texas A&M Health Science Center, College Station, Texas, United States of America; 6 Department of Food Science, Penn State University, University Park, Pennsylvania, United States of America; 7 The Penn State Hershey Cancer Institute, Hershey, Pennsylvania, United States of America; Instituto de Investigación Sanitaria INCLIVA, SPAIN

## Abstract

Limited information is available regarding the metabolic consequences of intestinal dysbiosis in dogs with acute onset of diarrhea. The aim of this study was to evaluate the fecal microbiome, fecal concentrations of short-chain fatty acids (SCFAs), as well as serum and urine metabolites in healthy dogs (n=13) and dogs with acute diarrhea (n=13). The fecal microbiome, SCFAs, and serum/urine metabolite profiles were characterized by 454-pyrosequencing of the 16S rRNA genes, GC/MS, and untargeted and targeted metabolomics approach using UPLC/MS and HPLC/MS, respectively. Significantly lower bacterial diversity was observed in dogs with acute diarrhea in regards to species richness, chao1, and Shannon index (p=0.0218, 0.0176, and 0.0033; respectively). Dogs with acute diarrhea had significantly different microbial communities compared to healthy dogs (unweighted Unifrac distances, ANOSIM p=0.0040). While Bacteroidetes, *Faecalibacterium*, and an unclassified genus within Ruminococcaceae were underrepresented, the genus *Clostridium* was overrepresented in dogs with acute diarrhea. Concentrations of fecal propionic acid were significantly decreased in acute diarrhea (p=0.0033), and were correlated to a decrease in *Faecalibacterium* (ρ=0.6725, p=0.0332). The predicted functional gene content of the microbiome (PICRUSt) revealed overrepresentations of genes for transposase enzymes as well as methyl accepting chemotaxis proteins in acute diarrhea. Serum concentrations of kynurenic acid and urine concentrations of 2-methyl-1H-indole and 5-Methoxy-1H-indole-3-carbaldehyde were significantly decreased in acute diarrhea (p=0.0048, 0.0185, and 0.0330, respectively). These results demonstrate that the fecal dysbiosis present in acute diarrhea is associated with altered systemic metabolic states.

## Introduction

Recent molecular studies have greatly increased our knowledge about the microbiota in the gastrointestinal tract (GIT) of dogs, mice, and humans [1–4]. The GIT microbiota plays an important role in host health by stimulating the immune system, influencing gut structure, aiding in the defense against pathogens, and providing nutritional benefits to the host (e.g., production of SCFAs) [5–10]. Despite recent advances in characterizing microbial communities using sequencing technology, there remains a rudimentary understanding of the complex interactions that occur between the host and intestinal microbes and their metabolic end-products. The analysis of host and bacterial metabolites may give additional insights into the pathophysiology of gastrointestinal diseases, including acute diarrhea. Metabolomics is a relatively new field that aims to characterize qualitatively and/or quantitatively the presence of small molecules in biological samples [11]. This approach may enhance our understanding of the host-microbe interactions, as well as the metabolic pathways that are involved in health and disease. Initial metagenomic and metabolomic studies performed in humans and animal models suggest that the metabolites derived from diverse microbial communities may have a direct role in health and disease [12]. Therefore, more in-depth studies are needed to understand the relationships between the microbiome and the host [8]. Phylogenetic changes in intestinal microbiota have been previously described in dogs with acute and chronic GI disease [13–15]. A previous study by our group has compared the fecal microbiome between healthy dogs, dogs with IBD, and dogs with acute diarrhea, but reported only phylogenetic data based on 16S rRNA gene sequences and it is currently unknown whether these microbiome changes are associated with metabolic changes in the host [14]. Therefore, the aim of the present study was to evaluate a new cohort of dogs with acute diarrhea to confirm the previous findings, and also to elucidate whether microbiome changes based on 16S rRNA genes are associated with metabolic and functional changes. We profiled the fecal microbiome using 16S rRNA sequencing, measured and correlated fecal metabolic end products (i.e., SCFAs) with bacterial groups, and inferred metagenomics using PICRUSt [16]. Furthermore, this study used Ultra Performance Liquid Chromatography-Mass Spectrometry (UPLC-MS) in an untargeted metabolomics approach coupled with High Performance Liquid Chromatography-Mass Spectrometry (HPLC-MS) in a targeted metabolomics approach to characterize and confirm metabolomic alterations in the serum and urine of dogs with acute diarrhea.

## Materials and Methods

### Animal enrollment and sample collection

Naturally passed feces, serum samples (collected by venipuncture), and urine samples (collected by cystocentesis) were obtained from healthy dogs as well as dogs with acute diarrhea (AD). Dogs with AD were further classified as having non-hemorrhagic diarrhea (NHD) or hemorrhagic diarrhea (AHD) (Table 1). Feces were refrigerated immediately after collection, transferred within a few hours to a -80°C freezer, and stored frozen until processing for DNA extraction. Serum and urine samples were aliquoted and stored frozen at -80°C until processing. Owners provided written consent for their dogs to be used in this study. The collection of feces, serum, and urine was approved by the Texas A&M University Institutional Animal Care and Use Committee (IACUC): Protocol Number; 2012–101. None of the healthy dogs or dogs with acute diarrhea had been used in a previous study [14].

**Table 1:** Summary of basic characteristics and alpha diversity measures.

	Healthy	NHD	AHD	AD	p-value
**Age (years; median, range)**	5.0, 1.0–12.0	1.0, 1.0–12.0	4.0, 1.0–10.0	3.0, 1.0–12.0	0.3988
**Weight (kg; median, range)**	20.4, 2.7–31.8	20.9, 7.3–31.6	16.9, 4.9–44.4	20.9, 4.9–44.4	0.7776
**Sex (female/male)**	8/5	4/1	2/4	6/5	0.9166
**OTU**_**97**_ **(mean ± SD)**	268.1^a^ ± 52.8	200.3 ± 48.5	204.6 ± 35.8	201.3^b^ ± 56.7	**0.0218**
**Shannon Index (mean ± SD)**	4.8^a^ ± 0.4	4.0^b,c^ ± 0.2	3.9^a,b,c^ ± 0.8	4.0^c^ ± 0.6	**0.0033**
**Chao1 (mean ± SD)**	386.1^a^ ± 80.6	291.2 ± 57.7	305.8 ± 109.7	303.6^b^ ± 87.0	**0.0176**

NHD = acute non-hemorrhagic diarrhea; AHD = acute hemorrhagic diarrhea; AD = both groups combined (NHD and AHD).

Means not sharing a common superscript are significantly different (p<0.05 based on Dunn’s multiple comparisons test).

The control group consisted of 13 healthy pet dogs (Table 1, S1 Table). All dogs were privately owned, lived in various home environments, and were fed a variety of commercial diets. None of the dogs had a history of gastrointestinal signs or administration of antibiotics for at least a month prior to collection of fecal samples. All healthy dogs lived in Texas, USA.

The diseased group consisted of 13 dogs in total with acute diarrhea (6 dogs with NHD and 7 dogs with AHD) that presented to the Veterinary Medical Teaching Hospital at Texas A&M University with acute, non-hemorrhagic or hemorrhagic diarrhea (defined as duration of diarrhea <3 days) (Table 1, S1 Table). None of the dogs had a previous history of GI signs or had received antibiotics within the previous three months. Diagnostic evaluation included a complete blood count (CBC), serum chemistry profiles (SIRRUS Clinical Chemistry Analyzer), serum concentrations of canine trypsin-like immunoreactivity (cTLI), serum concentrations of canine pancreatic lipase immunocreactivity (cPLI), serum concentrations of cobalamin and folate (Immulite 2000 Vitamin B12, Folic Acid, Siemens Medical Solutions Diagnostics), and serum concentrations of C-reactive protein (CRP; Phase Range Canine C-reactive Protein Assay, Tridelta Development Ltd), and urine analysis. None of the dogs with acute diarrhea had been readmitted to the clinic at Texas A&M University after one month of initial presentation and for the purpose of this study their clinical signs were considered resolved.

### DNA isolation

100 mg of feces were aliquoted into a sterile 1.7 ml tube (Microtube, Sarstedt AG & Co, Nümbrecht, Germany) containing 150 μl of 0.1 mm zirconia-silica beads and 100 μl of 0.5 mm zirconia-silica beads (BioSpec Products Inc., Barlesville, OK, USA). Samples were then homogenized (FastPrep-24, MP Biomedicals, USA) for a duration of 1 minute at a speed of 4 m/s. DNA was then extracted with the ZR fecal DNA Mini Prep kit following the manufacturer’s instructions (Zymo Research, Irvine CA, USA).

### 454-Pyrosequencing

Bacterial tag-encoded FLX-titanium amplicon pyrosequencing was performed targeting the V4–V6 region of the 16S rRNA gene using forward and reverse primers: 530F (5’-GTGCCAGCMGCNGCGG-3’) and 1100R (5’-GGGTTNCGNTCGTTG-3’), respectively [17]. Raw sequence data were screened, trimmed, de-noised, filtered, and depleted of chimeras using the QIIME v1.7 open-source pipeline [18]. Operational taxonomic units (OTUs) were assigned based on at least 97% sequence similarity using QIIME. The sequences were deposited in the Sequence Read Archive under the following accession number: SRP040310.

### Quantitative PCR (qPCR)

To evaluate specific bacterial groups of interest to intestinal health (i.e., *Lactobacillus* and *Bifidobacterium*) and bacterial species with potential pathogenic roles (i.e., *Escherichia coli*, and *Clostridium perfringens*) qPCR was used as described previously [19–21] and primers/probe can be viewed in the S4 Table. Quantitative PCR reactions were performed using two reaction chemistries. For a subset of assays SYBR-green based reaction mixtures were used, with a total reaction volume of 10 μl. The final mix contained 5 μl SsoFast EvaGreen supermix (Bio-Rad Laboratories, CA, USA), 0.4 μl each of a forward and reverse primer (final concentration: 400 nM), 2.6 μl of high quality PCR water, and 2 μl of normalized DNA (final concentration: 5 ng/μl). Conditions for PCR were as follows: initial denaturation at 98°C for 2 min, then 40 cycles with denaturation at 98°C for 3 sec and annealing (S4 Table) for 3 sec. Post-amplification, a melt curve analysis was performed using these conditions: 95°C for 1 min, 55°C for 1 min, and increasing incremental steps of 0.5°C for 80 cycles for 5 sec each. All samples were run in duplicate fashion. TaqMan based reaction mixtures were used in a total reaction volume of 10 μl. The final mix contained 5 μl TaqMan Fast Universal PCR master mix (Life Technologies, NY, USA), 0.4 μl of a forward and reverse primer (final concentration: 400 nM), 2 μl of high quality PCR water, and 2 μl of normalized DNA (final concentration: 5 ng/μl). Conditions for PCR were as follows: initial denaturation at 95°C for 20 sec then 40 cycles with denaturation at 95°C and annealing (S4 Table) for 3 sec. Post-amplification, a melt curve analysis was performed using these conditions: 95°C for 1 min, 55°C for 1 min, and increasing incremental steps of 0.5°C for 80 cycles for 5 sec each. All samples were run in duplicate fashion.

### Measurement of short-chain fatty acids

Analysis of short-chain fatty acids (SCFAs; i.e., acetate, propionate, and butyrate), and branched chain fatty acids (BCFAs; i.e., isobutyrate, isovalerate, and valerate) in feces was performed on a subgroup of dogs, as not all dogs had sufficient quantity of feces available. The SCFA and BCFA were measured using a dilution gas chromatography-mass spectrometry (GC-MS) assay as previously described [22] with some modifications. Briefly, the fecal samples were weighed and diluted 1:5 in extraction solution (2N hydrochloric acid). After homogenization using a multi-tube vortexer for 30 min at room temperature, fecal suspensions were centrifuged for 20 min at 2,100 x g and 4°C. Supernatants were then collected using serum filters (Fisherbrand serum filter system, Fisher Scientific Inc, Pittsburgh, Pa). From each sample, 500 μl of supernatant were mixed with 10 μl of internal standard (200 mM heptadeuterated butyric acid) and extracted using a C18 solid phase extraction column (Sep-Pak C18 1 cc Vac Cartridge, Waters Corporation, Milford, MA). Samples were derivatized using *N*-tert-butyldimethylsilyl-*N*-methyltrifluoroacetamide (MTBSTFA) at room temperature for 60 min. A gas chromatographer (Agilent 6890N, Agilent Technologies Inc, Santa Clara, CA) coupled with a mass spectrometer (Agilent 5975C, Agilent Technologies Inc, Santa Clara, CA) was used for chromatographic separation and quantification of the derivatized samples. Separation was achieved using a DB-1ms capillary column (30 m x 0.25 mm, 0.25 μm film thickness, Agilent Technologies Inc, Santa Clara, CA). The GC temperature program was as follows: 40°C held for 0.1 min, increased to 70°C by 5°C/min, 70°C held for 3.5 min, increased to 160°C by 20°C/min, and finally increased to 280°C for 3 min by 35°C/min. The total run time was 20.53 min. Solvent delay was 5 min. The mass spectrometer was operated in electron impact positive-ion mode with selective ion monitoring at mass-to-charge ratios (*M/Z*) of 117 (acetate), 131 (propionate), 145 (butyrate and isobutyrate), 152 (deuterated butyrate; internal standard), and 159 (valerate and isovalerate). The estimate of the relative concentrations was based on the ratio of the area under the curve of the internal standard for each fatty acid.

### Measurement of dry weight

For determination of fecal dry weight, a 100 mg of feces from each sample was aliquoted into a sterile 1.7 ml serum tube (Microtube, Sarstedt AG & Co, Nümbrecht, Germany). This aliquot was solely used in the determination of dry weight and thus was weighed and dried at 105°C in an oven (Symphony Gravity Convection Oven, VWR) overnight. The percent of dry weight was calculated and SCFA/BCFA concentrations were adjusted (measured in μmol/g of dry feces for all SCFAs/BCFAs) to normalize by dry weight.

### Untargeted metabolite detection in serum

Serum samples were thawed on ice and diluted 1:5 in cold methanol. The mixture was vortexed and incubated at -20°C for two hours to precipitate protein. The solution was centrifuged at 14,000 x g for 20 minutes at 4°C, and then the supernatant was collected and transferred to a Waters 96 well autosampler plate. 1 μl injections were performed on a Waters Acquity UPLC system. Separation was performed using a Waters Acquity UPLC C8 column (1.8 μm, 1.0 x 100 mm), using a gradient from solvent A (95% water, 5% methanol, 0.1% formic acid) to solvent B (95% methanol, 5% water, 0.1% formic acid). Injections were made in 100% A, which was held for 0.1 min, ramped to 40% B in 0.9 min, to 70% B over two min, and to 100% B over 8 min. Mobile phase was held at 100% B for 6 min, returned to starting conditions over 0.1 min, and allowed to re-equilibrate for 5.9 min. Flow rate was constant at 140 μl/min for the duration of the run. The column was held at 50°C and samples were held at 10°C. Column Eluent was infused into a Waters Xevo G2 Q-TOF MS fitted with an electrospray source. Data was collected in positive ion mode, scanning from 50–1200 at a rate of 0.2 sec per scan. Collision energy was set to 6 Volts for MS mode, and ramped from 15 to 30 volts for MSE mode. Calibration was performed prior to sample analysis via infusion of sodium formate solution, with mass accuracy within 1 ppm. The capillary voltage was held at 2200 volts, the source temp at 150°C, and the desolvation temperature at 350°C at a nitrogen desolvation gas flow rate of 800 L/hr.

### Untargeted metabolite detection in urine

Analysis of metabolites was performed on a subgroup of dogs, as urine was unable to be collected from all dogs. Urine samples were diluted 1:2 in methanol, centrifuged, and transferred to a Waters 96 well autosampler plate. The peak areas were first normalized based on total extracted ion signal and then normalized based on urine creatinine concentrations measured by a SIRRUS Clinical Chemistry Analyzer. A volume of 5 μl was injected into a Waters Acquity UPLC system. Separation was performed using a Waters Acquity UPLC T3 column (1.8 μm, 1.0 x 100 mm), using a gradient from solvent A (water, 0.1% formic acid) to solvent B (acetonitrile, 0.1% formic acid). Injections were made in 100% A, which was held for 1 min, a 12 min linear gradient to 95% B was applied, and held at 95% B for 3 min, returned to starting conditions over 0.05 min, and allowed to re-equilibrate for 3.95 min. Flow rate was constant at 200 μl/min for the duration of the run. The column was held at 50°C and the samples were held at 5°C. Column eluent was infused into a Waters Xevo G2 Q-TOF MS fitted with an electrospray source. Data was collected in positive ion mode, scanning from 50–1200 at a rate of 0.2 sec per scan, alternating between MS and MSE mode. Collision energy was set to 6 Volts for MS mode, and ramped from 15 to 30 volts for MSE mode. Calibration was performed prior to sample analysis via infusion of sodium formate solution, with mass accuracy within 1 ppm. The capillary voltage was held at 2200V, the source temp at 150°C, and the desolvation temperature at 350°C at a nitrogen desolvation gas flow rate of 800 L/hr.

### Targeted metabolite detection in serum and urine

Standards for Kynurenic Acid (IUPAC: 4-hydroxyquinooline-2-carboxyclic acid) and 2-methylindole were purchased from Sigma-Aldrich and were ≥98% pure. The standard for 5-Methoxy-1H-indole-3-carbaldehyde was purchased from Matrix Scientific and was ≥98% pure. The target compounds in samples were detected and quantified on a triple quadrupole linear ion trap mass spectrometer (3200 QTRAP, AB SCIEX, Foster City, CA) coupled to a binary pump HPLC (Prominence LC-20, Shimazu, Concord, Ontario, Canada). Peak identification and integration were performed using Analyst software (version 5, Agilent, Foster City, CA). Samples were maintained at 4°C on an autosampler prior to injection. Chromatographic separation was achieved on a C-18 column (Synergi Fusion 4 μm 80 Å 150 mm × 2 mm, Phenomenex, Torrance, CA) using a solvent gradient method [23]. Solvent A was formic acid solution in water (0.1%). Solvent B was acetonitrile with formic acid (0.1%). Injection volume was 10 μL. For urine, concentrations of compound within each urine sample were normalized to urine creatinine concentration and expressed as ratios. Serum was normalized by volume.

### Statistical analysis

A subset of 6,900 sequences per sample was randomly selected to account for unequal sequencing depth across samples. Differences in microbial communities between healthy dogs, dogs with NHD, and dogs with AHD were analyzed using the phylogeny-based unweighted UniFrac distance metric, PCoA plots, and rarefaction curves generated by QIIME [18]. Rarefaction curves and PCoA plots show alpha (i.e., Chao 1, Shannon Index, and Observed Species) and beta (i.e., microbial community distance matrix) diversity, respectively. ANOSIM (Analysis of Similarity) within the software package PRIMER 6 (PRIMER-E Ltd., Luton, UK) was used to determine significant differences in microbial communities between healthy dogs and diseased dogs. To visualize the relative abundance of bacterial families for individual fecal samples, heat maps were generated in NCSS 2007 (NCSS, Kaysville, Utah).

All datasets were tested for normality using the Shapiro-Wilk test (JMP 10, SAS software Inc.). Because most datasets did not meet the assumptions of normal distribution, comparisons between healthy and disease groups were determined using non-parametric Kruskal-Wallis tests (healthy dogs vs. dogs with NHD vs. dogs with AHD) or a Mann-Whitney U test (healthy dogs vs. dogs with acute diarrhea AD [dogs with NHD and dogs with AHD combined]). Taxa that were present in at least 70% of dogs (either healthy or diseased) were included in 454-pyrosequencing data analysis. The resulting p-values of the Kruskal-Wallis tests or Mann-Whitney U test were adjusted for multiple comparisons using the Benjamini & Hochberg’s False Discovery Rate (FDR), and an adjusted p<0.05 was considered statistically significant [24]. A Dunn’s post-test was used to determine which disease types were significantly different if applicable.

### PICRUSt

The software PICRUSt (Phylogenetic Investigation of Communities by Reconstruction of Unobserved States) was used to make functional gene content predictions based on 16S rRNA gene data present in the Greengenes database [25]. PICRUSt is freely available online in the Galaxy workflow framework and can also be used through the QIIME open-source pipeline [26, 27].

### LEfSe

Linear discriminant analysis effect size (LEfSe) was used to elucidate taxa and genes associated with healthy or diseased states. For bacterial groups, the LDA score threshold was set to > 3.5; for functional genes and their specific KEGG orthologs the LDA score threshold was set to > 2.5. LEfSe is freely available online in the Galaxy workflow framework [26, 27].

### Short-chain fatty acids

Fecal SCFA concentrations were tested for normality and compared between each group using a Mann-Whitney U test. SCFAs that were found to be significantly different between groups were then correlated to differentially abundant bacterial groups (i.e., Spearman’s rank correlation; JMP 10, SAS software Inc.).

### Serum and urine untargeted metabolomics

Waters raw files were converted to netCDF format, and XCMS [28] peak detection, retention time alignment, and feature grouping were performed on both the low and high collision energy channels (MS and MSe). The datasets were separated following alignment, normalized to total extracted ion signal, averaged by injection replicate, and the idMS/MS workflow described previously was applied to all significant features for generation of indiscriminant MS/MS (idMS/MS) spectra for library searching and compound identification [29]. For identification of spectra, recreated idMS/MS spectra were searched against the NIST ‘12 MS/MS database, Metlin, and Massbank. idMS/MS spectra which matched database MS/MS spectra were assigned a level 2 identification confidence [30]. For urine, concentrations of compound within each urine sample were normalized to urine creatinine concentration and expressed as ratios. Serum was normalized by volume. After data pre-processing, principle component analysis and univariate analysis was conducted using the web-based metabolomic data processing tool MetaboAnalyst 2.0 [31, 32].

## Results

### Sequencing analysis

**Fig 1:**
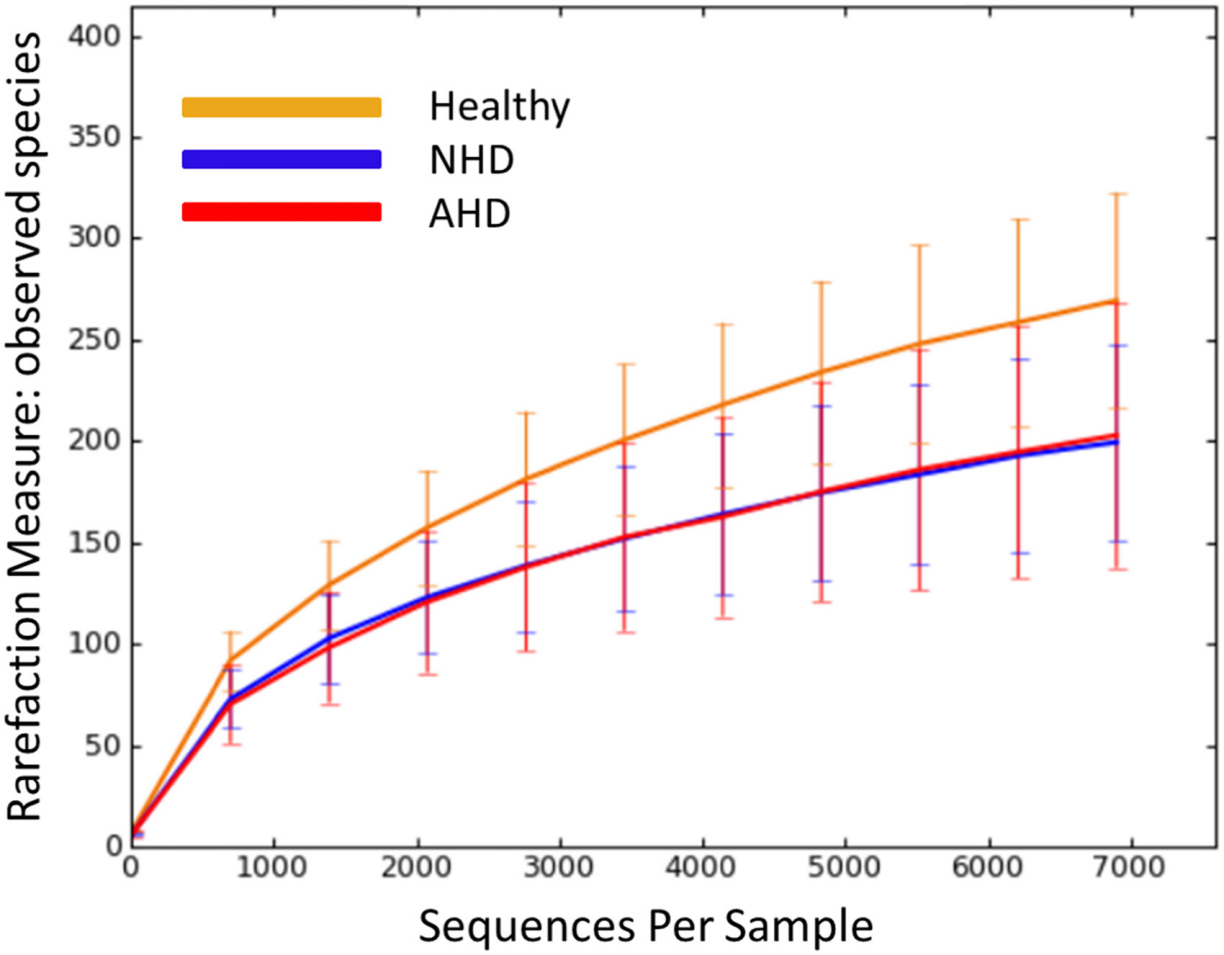
Rarefaction analysis of 16S rRNA gene sequences obtained from canine fecal samples. Lines represent the mean and error bars represent standard deviations. The analysis was performed on a randomly selected subset of 6,900 sequences per sample. NHD = acute non-hemorrhagic diarrhea; AHD = acute hemorrhagic diarrhea.

The analysis pipeline yielded 297,315 quality sequences for the 19 samples analyzed (mean ± standard deviation [of all samples] = 9,013 ± 1,203). Fig 1 illustrates the rarefaction curve for observed species (a count of all unique operational taxonomic units (OTUs)). All alpha diversity measures are summarized in Table 1. Observed species were significantly decreased in dogs with acute diarrhea (p = 0.0218). The Shannon Index was significantly decreased in dogs with AD and NHD compared to healthy dogs (p = 0.0033 and p < 0.0500, respectively). The Chao1 predictive diversity measure was also significantly decreased in dogs with acute diarrhea (p = 0.0176).

### Microbial communities

**Fig 2:**
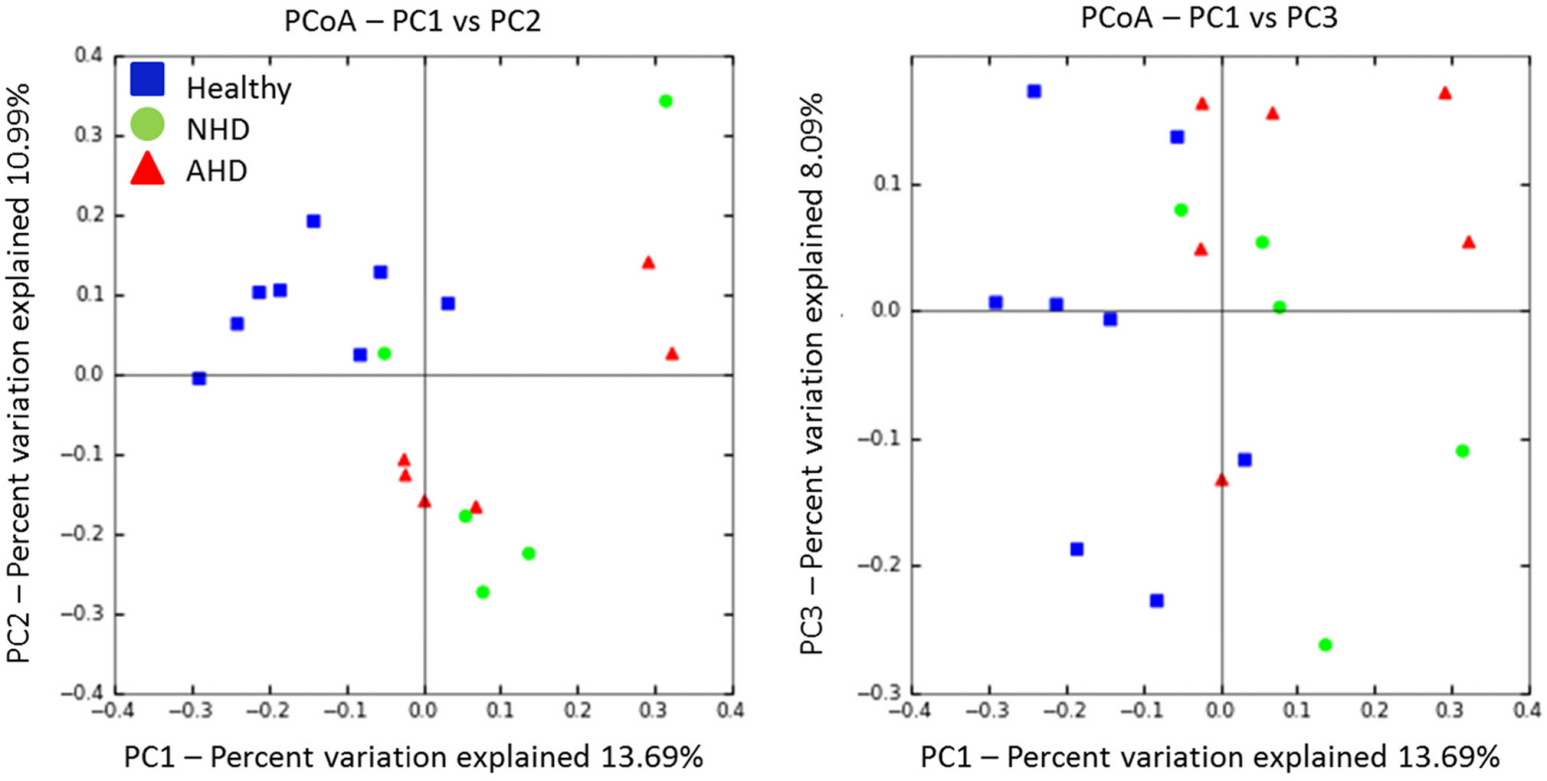
Principal Coordinate Analysis (PCoA) of unweighted UniFrac distances of 16S rRNA genes. Blue squares = healthy dogs, green circles = acute non-hemorrhagic diarrhea (NHD), and red triangles = acute hemorrhagic diarrhea (AHD); ANOSIM for healthy dogs vs. dogs with AD (NHD and AHD combined), p = 0.0040; and ANOSIM for NHD or AHD vs. healthy dogs, p = 0.0020 for both.

**Fig 3:**
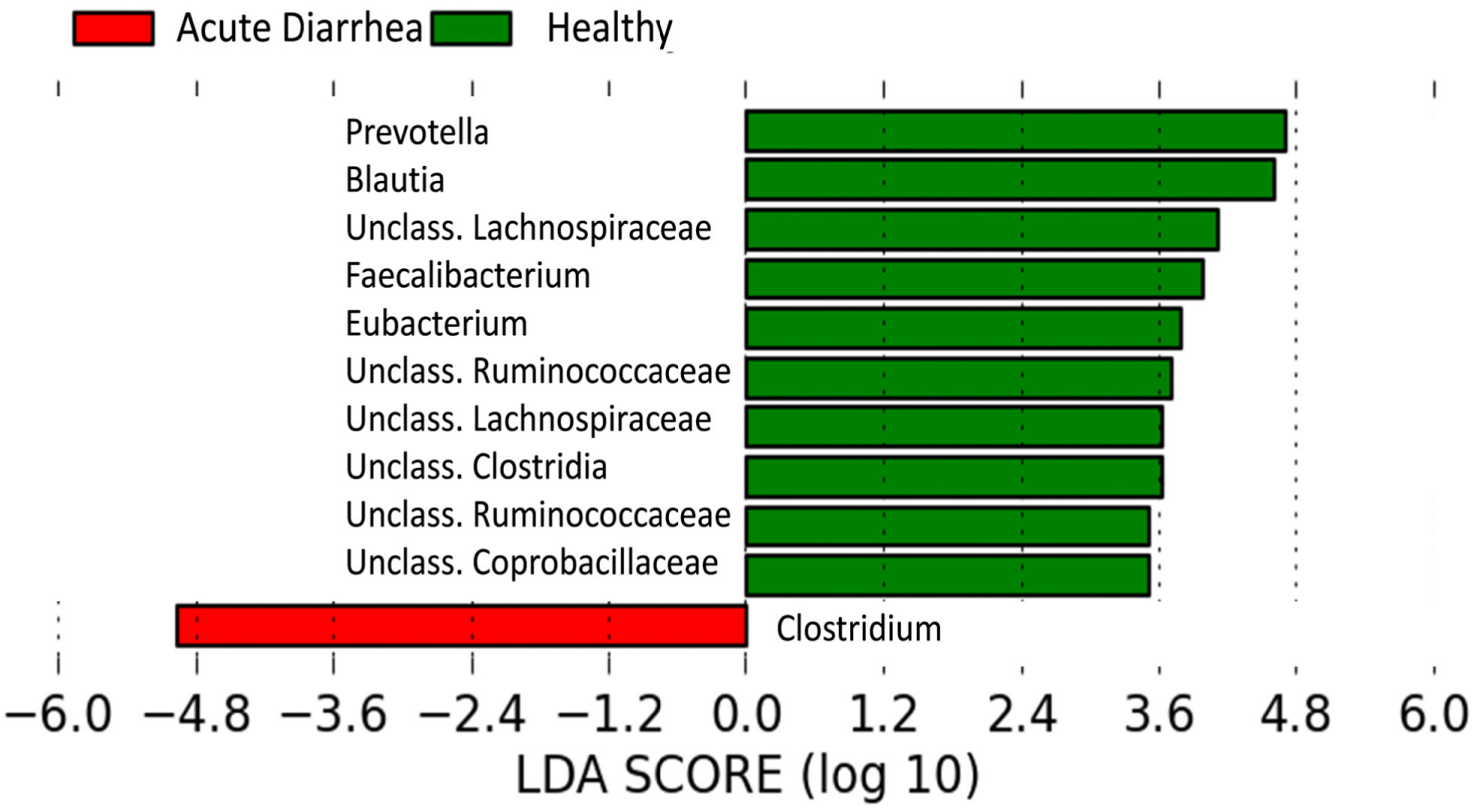
Differentially abundant bacterial groups. Groups differentially abundant between healthy dogs and dogs with acute diarrhea. Red bars represent bacterial groups associated with dogs with acute diarrhea, while green bars represent bacterial groups associated with healthy dogs.

**Fig 4:**
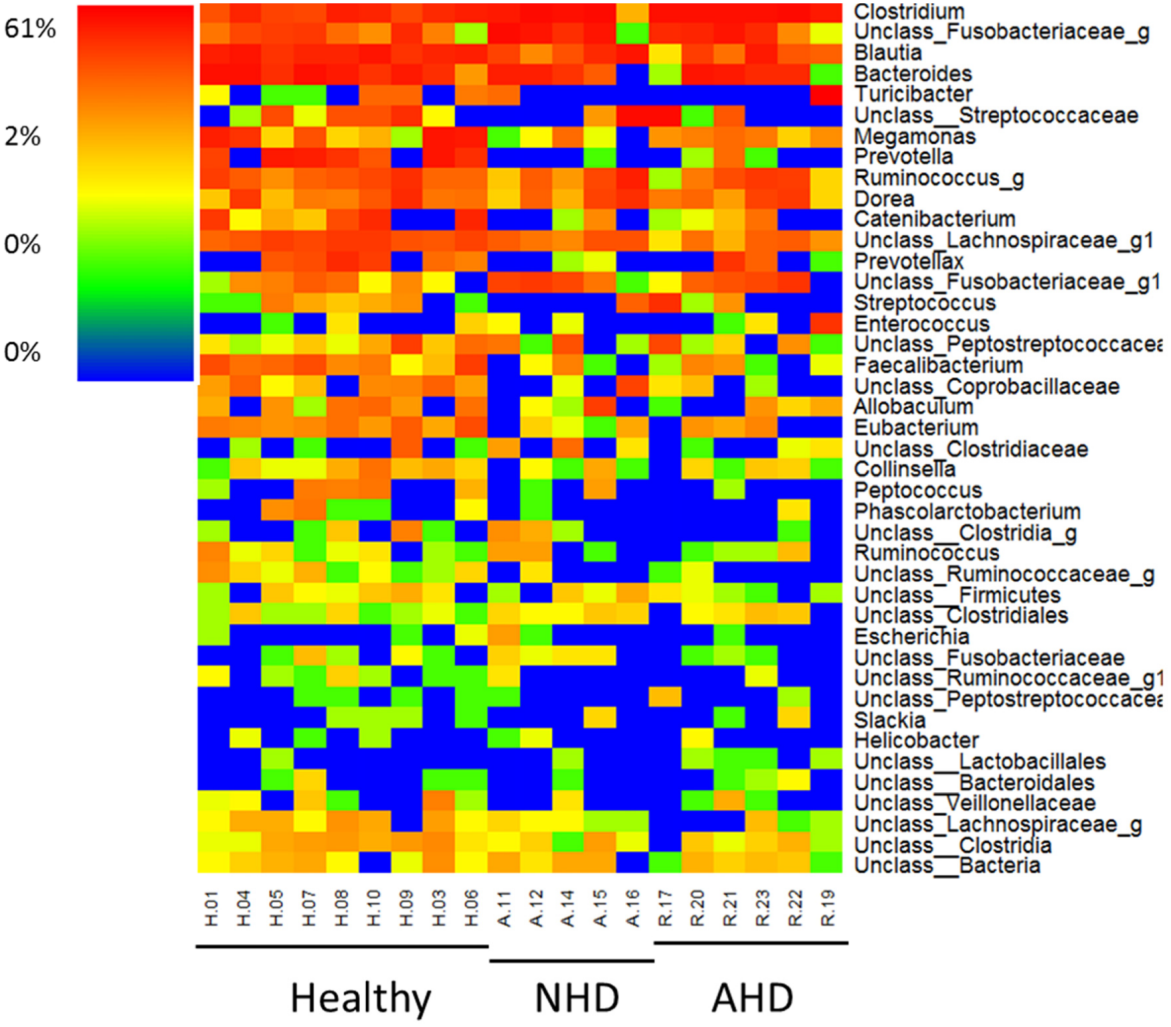
Heatmap illustrating the relative abundance of predominant bacterial genera in fecal samples. Healthy = healthy dogs; NHD = acute non-hemorrhagic diarrhea; AHD = acute hemorrhagic diarrhea. “Unclass.” denotes an unclassified genus within the respective taxa.

PCoA plots (Fig 2) based on unweighted Unifrac distances showed significant differences between healthy dogs and dogs with AD (ANOSIM; p = 0.0040). Furthermore dogs with NHD and dogs with AHD differed significantly from healthy dogs (ANOSIM; p = 0.0020 for both). There was no difference in microbial communities, however, between dogs with hemorrhagic and non-hemorrhagic diarrhea. Based on LDA effect size (LEfSe), *Clostridium* spp. was significantly associated with AD, while *Prevotella* spp., *Blautia* spp., *Faecalibacterium* spp., *Eubacterium* spp., and unclassified genera within the following families: Ruminococcaceae, Lachnospiraceae, Clostridia, Ruminococcaceae, and Coprobacillaceae were significantly associated with healthy dogs (Fig 3). A heatmap in Fig 4 was employed to illustrate these changes.

**Fig 5:**
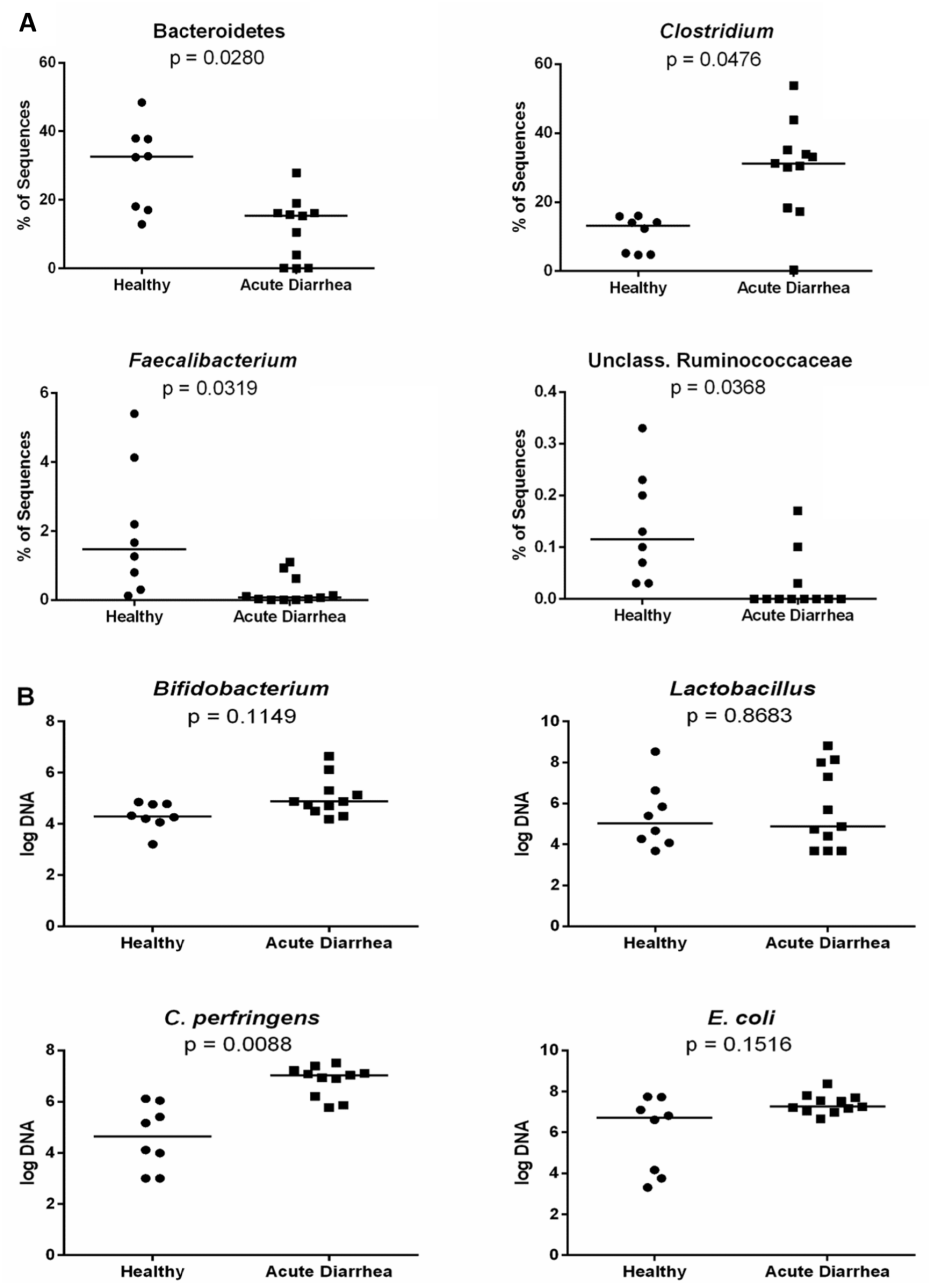
Groups of interest based on A) Sequencing (% of sequences) and B) quantitative PCR results. The qPCR data was expressed as log amount of DNA for each particular bacterial group per 10 ng of isolated total DNA. Acute Diarrhea = both groups combined (NHD and AHD). Bars represent the median value for each group. P-values adjusted based on the Benjamini and Hochberg false discovery rate.

Table 2 and Fig 5A summarize differences in bacterial groups between disease groups. Sequences belonging to the phylum Bacteroidetes were significantly decreased in dogs with AD compared to healthy dogs (p = 0.0280). Sequences belonging to the genus *Faecalibacterium* and an unclassified genus within Ruminococcaceae were both significantly decreased in dogs with AD compared to healthy dogs (p = 0.0319 and 0.0368, respectively). Sequences belonging to the genus *Clostridium* were significantly increased in dogs with AD compared to healthy dogs (p = 0.0476). No differences were identified between dogs with hemorrhagic and non-hemorrhagic acute diarrhea when evaluating bacterial groups.

**Table 2:** Percentages of the most abundant bacterial groups.

	median(min-max) *in percent		
Taxa	Healthy	Acute Diarrhea	p-value
**Phylum**			
Bacteroidetes	32.6(12.9–48.4)	15.3(0.0–27.9)	**0.0280**
Firmicutes	60.9(41.3–86.6)	72.2(32.7–99.9)	0.7213
Fusobacteria	4.5(0.1–12.7)	16.5(0.1–49.5)	0.0793
Proteobacteria	0.1(0.0–0.3)	0.1(0.0–1.2)	0.9596
unclass. bacteria	0.2(0.0–0.8)	0.3(0.0–0.4)	0.9008
**Genus**			
*Allobaculum*	0.6(0.0–1.9)	0.1(0.0–5.7)	0.4284
*Bacteroides*	14.2(0.6–32.7)	12.5(0.0–18.9)	0.4530
*Blautia*	14.0(8.9–25.2)	3.6(0.2–19.8)	0.0977
*Catenibacterium*	0.3(0.0–13.2)	0.1(0.0–1.6)	0.2893
*Clostridium*	13.2(4.7–16.0)	31.2(0.3–53.8)	**0.0476**
*Collinsella*	0.3(0.1–1.7)	0.0(0.0–0.4)	0.1058
*Dorea*	1.5(0.3–12.6)	2.0(0.2–9.8)	0.8286
*Enterococcus*	0.0(0.0–0.2)	0.0(0.0–9.2)	0.8810
*Escherichia*	0.0(0.0–0.1)	0.0(0.0–0.5)	0.9574
*Eubacterium*	1.0(0.4–3.8)	0.1(0.0–1.0)	0.0504
*Faecalibacterium*	1.5(0.1–5.4)	0.1(0.0–1.1)	**0.0319**
*Helicobacter*	0.0(0.0–0.1)	0.0(0.0–0.1)	0.8866
*J2-29*	0.8(0.0–2.8)	3.5(0.0–8.2)	0.2570
*Megamonas*	1.9(0.1–20.5)	0.6(0.0–1.9)	0.4043
*Peptococcus*	0.2(0.0–1.3)	0.0(0.0–0.5)	0.3683
*Phascolarctobacterium*	0.0(0.0–1.3)	0.0(0.0–0.2)	0.1953
*Prevotella*	9.5(0.0–25.1)	0.0(0.0–1.7)	0.0896
*[Prevotella]*	2.5(0.0–11.6)	0.0(0.0–7.8)	0.2358
*[Ruminococcus]*	2.7(0.7–10.6)	2.8(0.1–15.6)	0.8770
*Ruminococcus*	0.1(0.0–0.2)	0.0(0.0–0.5)	0.7934
*Slackia*	0.0(0.0–0.1)	0.0(0.0–0.2)	0.7038
*Streptococcus*	0.3(0.0–1.3)	0.0(0.0–9.7)	0.3614
*Turicibacter*	0.0(0.0–2.2)	0.0(0.0–61.1)	0.2956
unclass. bacteria	0.2(0.0–0.8)	0.3(0.0–0.4)	0.9228
unclass. Bacteroidales	0.0(0.0–0.2)	0.0(0.0–0.1)	0.8348
unclass. Clostridia_1	0.4(0.1–0.9)	0.1(0.0–0.5)	0.1098
unclass. Clostridia_2	0.0(0.0–1.1)	0.0(0.0–0.7)	0.8142
unclass. Clostridiaceae	0.0(0.0–2.7)	0.0(0.0–1.8)	0.8022
unclass. Clostridiales	0.1(0.0–0.3)	0.2(0.0–0.3)	0.3854
unclass. Coprobacillaceae	0.7(0.0–2.6)	0.0(0.0–5.4)	0.1159
unclass. Firmicutes	0.2(0.0–0.4)	0.1(0.0–0.4)	0.5854
unclass. Fusobacteriaceae_1	0.0(0.0–0.3)	0.0(0.0–0.2)	0.9080
unclass. Fusobacteriaceae_2	3.2(0.1–11.8)	12.0(0.0–44.0)	0.2534
unclass. Lachnospiraceae_1	4.7(3.2–6.7)	1.6(0.2–3.6)	0.0840
unclass. Lachnospiraceae_2	0.4(0.0–0.6)	0.1(0.0–0.3)	0.0905
unclass. Lactobacillales	0.0(0.0–0.1)	0.0(0.0–0.1)	0.3629
unclass. Peptostreptococcaceae_1	0.0(0.0–0.0)	0.0(0.0–0.3)	0.7815
unclass. Peptostreptococcaceae_2	0.3(0.1–5.7)	0.1(0.0–4.5)	0.4253
unclass. Ruminococcaceae_1	0.1(0.0–0.3)	0.0(0.0–0.2)	**0.0368**
unclass. Ruminococcaceae_2	0.0(0.0–0.2)	0.0(0.0–0.2)	0.1900
unclass. Streptococcaceae	1.8(0.0–10.8)	0.0(0.0–41.0)	0.3786
unclass. Veilonellaceae	0.1(0.0–1.1)	0.0(0.0–0.4)	0.4113

P-value adjusted based on the Benjamini and Hochberg False Discovery Rate.

The abbreviation "unclass." denotes an unclassified taxonomy within the respective taxonomic group.

### Quantitative PCR

The abundance of *Clostridium perfringens* was significantly increased in dogs with AD (log DNA median [range]: 7 [5.8–7.5]) compared to healthy dogs (log DNA median [range]: 4.6 [3–6.1]; p = 0.0088). *Clostridium perfringens* was also significantly increased in the subgroup dogs with AHD (log DNA median [range]: 7.1 [6.9–7.4]) compared to healthy dogs (p<0.0500). No significant difference in *Bifidobacterium*, *Lactobacillus*, or *E*. *coli* was identified between dogs with acute diarrhea and healthy dogs (Fig 5B).

### Functional genes

**Fig 6:**
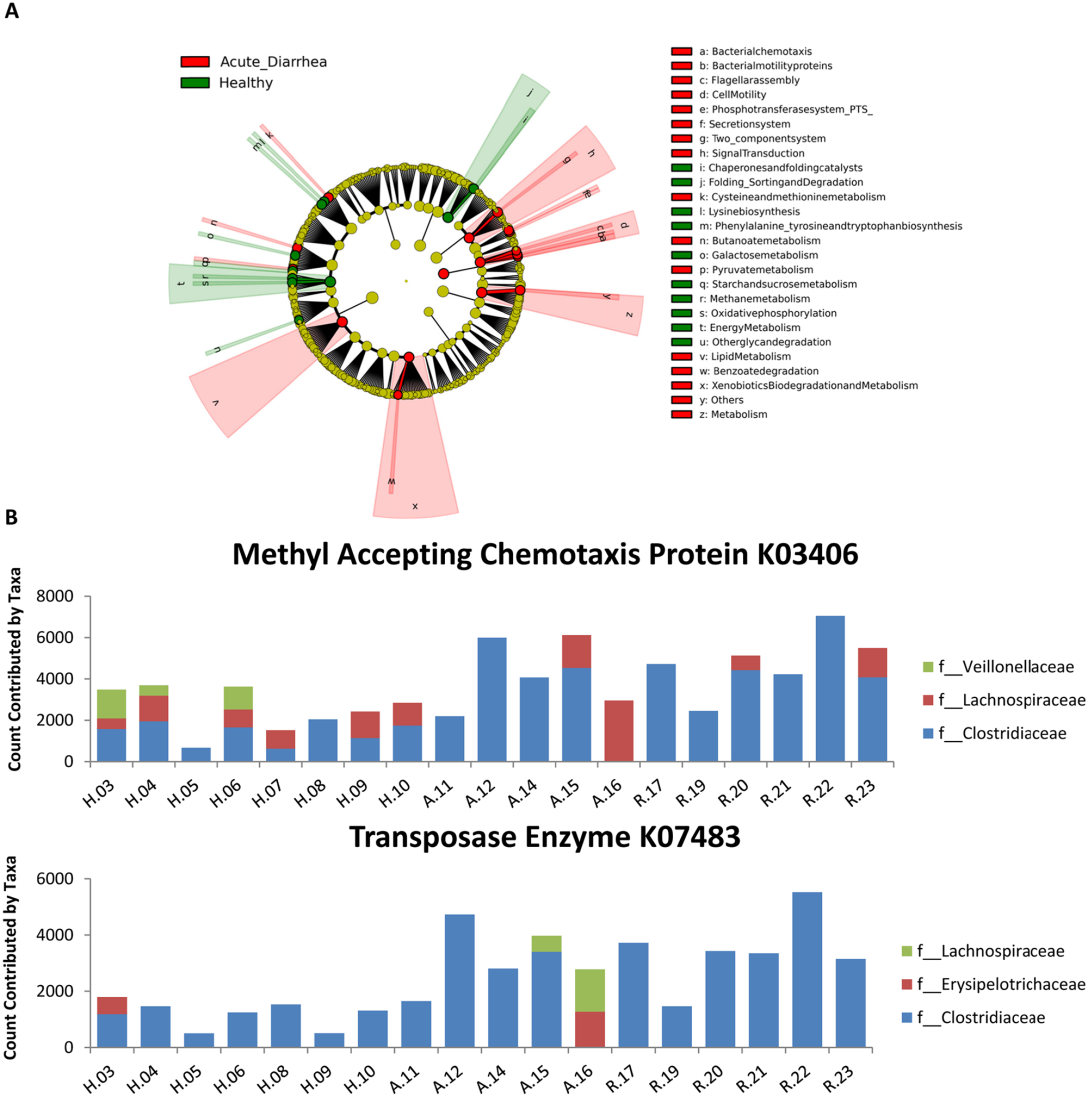
A) Differentially abundant gene families identified within healthy dogs and dogs with acute diarrhea. B) Taxa contributing to the MAC protein gene and Tranposase enzyme gene (K03406 and K07483, respectively) (Table 3) in all dogs. H.__ = Healthy, A.__ = acute non-hemorrhagic diarrhea, R.__ acute hemorrhagic diarrhea.

Univariate statistics revealed no significant differences in the percentage of KEGG orthologs belonging to functional gene families at all levels (e.g., 1, 2, and 3) among all groups of dogs after correcting for multiple comparisons (S2 Table). However, PICRUSt provided a snapshot of the distribution of genes across functional categories. At level 1, approximately 50% of genes belonged to metabolism, 19% belonged to genetic information processing, and 15% belonged to environmental information processing. Next, functional gene categories were analyzed using LEfSe. The cladogram in Fig 6A shows functional genes associated with healthy dogs and dogs with AD at all levels in the KEGG database hierarchy (LDA score threshold > 2.5). The following pathways were associated with acute diarrhea: benzoate degradation within xenobiotics biodegradation/metabolism, lipid metabolism, two component system within signal transduction, flagellar assembly/bacterial motility proteins/bacterial chemotaxis within cell motility, and metabolism. The following pathways were associated with healthy dogs: methane metabolism/oxidative phosphorylation within energy metabolism and chaperones/folding catalysts within folding sorting/degradation.

In a separate analysis, KEEG orthologs (KOs) were analyzed by LEfSe without being categorized by functional hierarchy (this strategy allows for more finite discovery of specific genes associated with healthy dogs or dogs with AD) (Table 3). KOs found to be associated with healthy dogs were K07133, K07720, K02027, K02026, K0205, K10439, and K07718. These genes are responsible for the multiple sugar transport system permease protein, multiple sugar transport system substrate-binding protein, ribose transport system substrate-binding protein, two-component system/sensor histidine kinase YesM, ATPase enzyme, and two-component system/response regulator YesN, respectively. KOs found to be associated with dogs with acute diarrhea were K03406 and K07483. These genes are responsible for the methyl-accepting chemotaxis protein and transposase enzyme, respectively. PICRUSt allows the user to determine which taxa contribute to functional gene predictions (Fig 6B). To gain an overall understanding of taxa contributing to identification of genes associated with acute diarrhea, only taxa contributing 500 or more gene counts were included in the bar charts. K03406 and K07483 were both identified as genes that were significantly associated with acute diarrhea. The charts in Fig 6B show that the family Clostridiaceae contributed mainly to genes coding for the methyl accepting chemotaxis protein as well as the transposase enzyme (K03406 and K07483, respectively). In addition, more gene counts appeared to be contributed by the family Clostridiaceae in dogs with acute diarrhea compared to healthy dogs.

**Table 3:** KEGG orthologs and functions that are highly abundant.

Group	KEGG ortholog	LDA score	Function
Healthy	K02025	2.9	multiple sugar transport system permease protein
Healthy	K02026	2.8	multiple sugar transport system permease protein
Healthy	K02027	2.8	multiple sugar transport system substrate-binding protein
Healthy	K10439	2.7	ribose transport system substrate-binding protein
Healthy	K07718	2.6	two-component system, sensor histidine kinase YesM
Healthy	K07133	2.6	ATPase
Healthy	K07720	2.5	two-component system, response regulator YesN
Acute Diarrhea	K03406	2.7	methyl-accepting chemotaxis protein
Acute Diarrhea	K07483	2.6	transposase

Performed by LEfSe using LDA score cutoff > 2.5.

### Short-chain fatty acids

**Fig 7:**
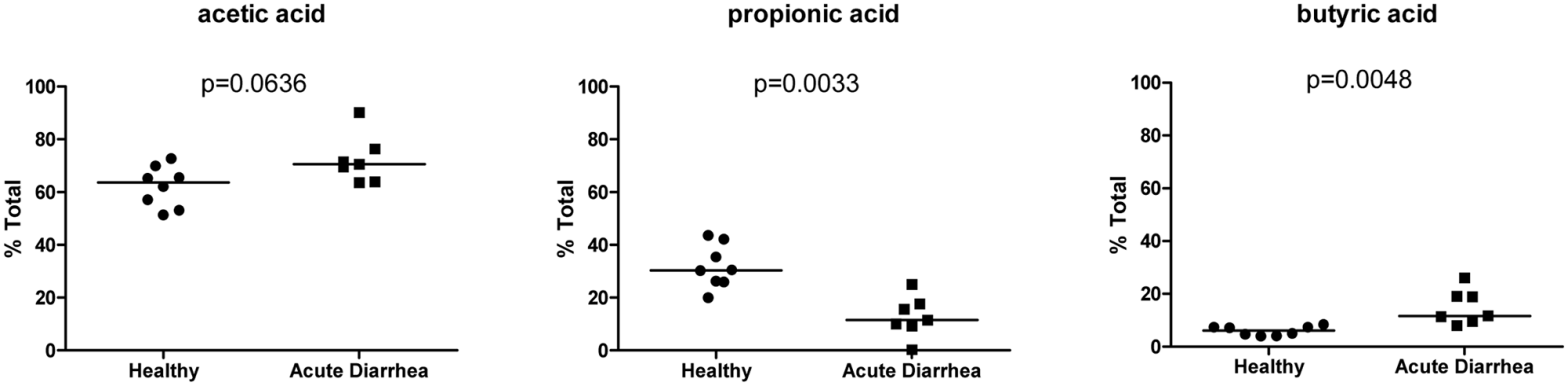
Proportion of total fecal SCFA concentrations. Circles = healthy dogs; Squares = acute diarrhea both groups combined (NHD and AHD). Bars represent the median value for each group. P-values adjusted based on the Benjamini and Hochberg false discovery rate.

Butyric acid, acetic acid, and propionic acid were each expressed as a percentage of the total amount of fecal SCFA concentrations (Fig 7). The proportion of propionic acid was significantly decreased in dogs with AD (median [range]:12% [0–25%]) compared to healthy dogs (median [range]: 30% [20–44%]; p = 0.0033). In contrast, the proportion of butyric acid was significantly increased in dogs with AD (median [range]: 12% [8–26%]) compared to healthy dogs (median [range]: 6% [4–8%]; p = 0.0048). There were no significant differences in total fecal SCFA or BCFA concentrations (μmol/g of dry feces) observed between groups of dogs (S3 Table).

### Correlations between SCFA and bacterial groups

**Fig 8:**
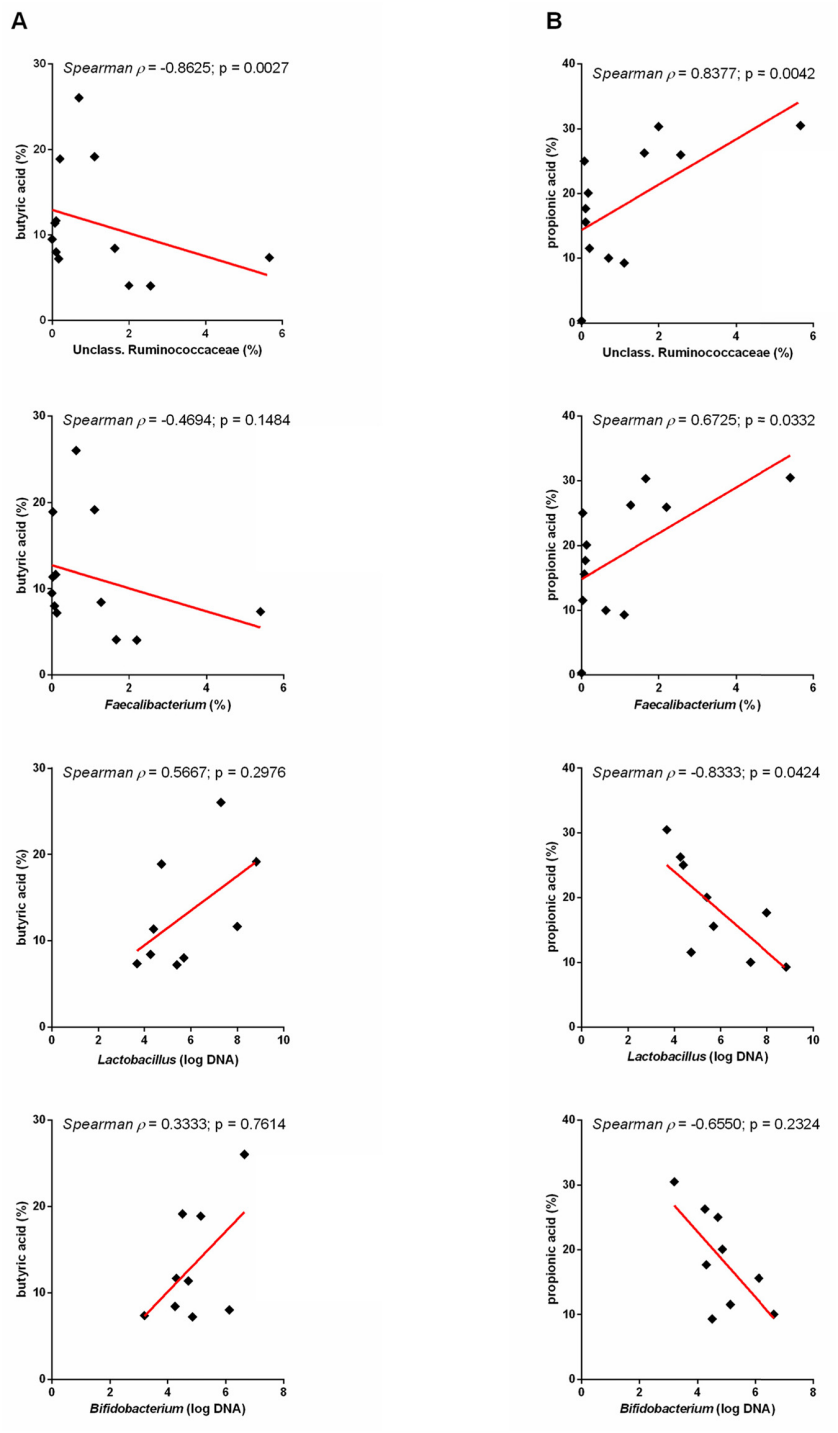
Correlations between SCFAs and bacterial groups. A) Correlations between butyric acid and select bacterial groups. B) Correlations between propionic acid and select bacterial groups.

A positive correlation was observed between propionic acid and an unclassified genus within Ruminococcaceae as well as *Faecalibacterium* (ρ = 0.8377 [p = 0.0042] and ρ = 0.6725 [p = 0.0332], respectively) (Fig 8). Butyric acid was found to have a negative correlation with an unclassified genus within Ruminococcaceae (ρ = -0.8265 [p = 0.0027]. Furthermore, propionic acid was negatively correlated with *Lactobacillus* (ρ = -0.8333 [p = 0.0424]).

### Serum and urine metabolites

**Fig 9:**
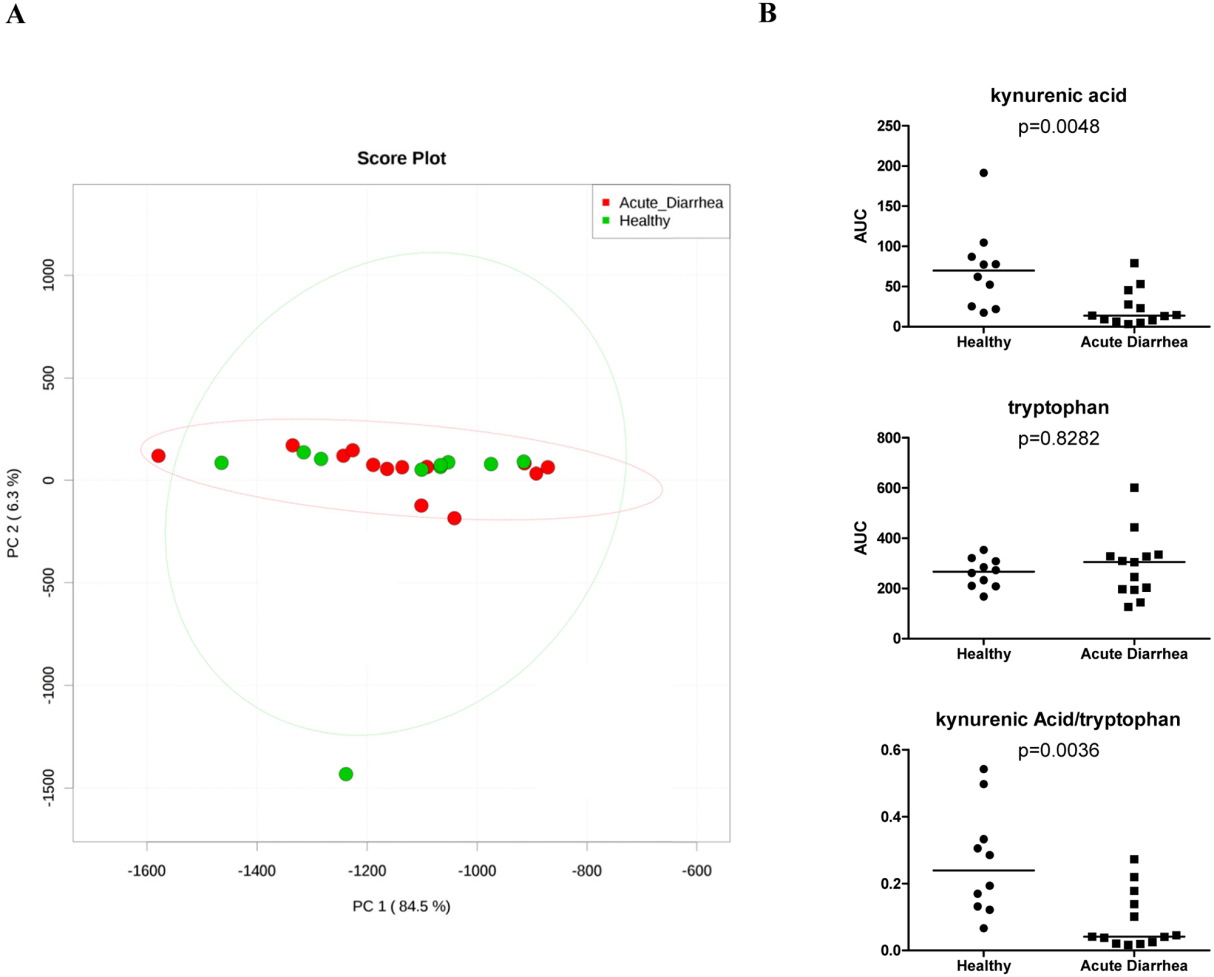
Analysis of serum metabolites. A) Principle component analysis plot where red dots represent dogs with acute diarrhea while green dots represent healthy dogs. Ellipses show the 95% confidence distribution for each group. B) Relative concentration of kynurenic acid, tryptophan, and their ratio to one another in serum (p = 0.0048, 0.8282, and 0.0036; respectively).

**Fig 10:**
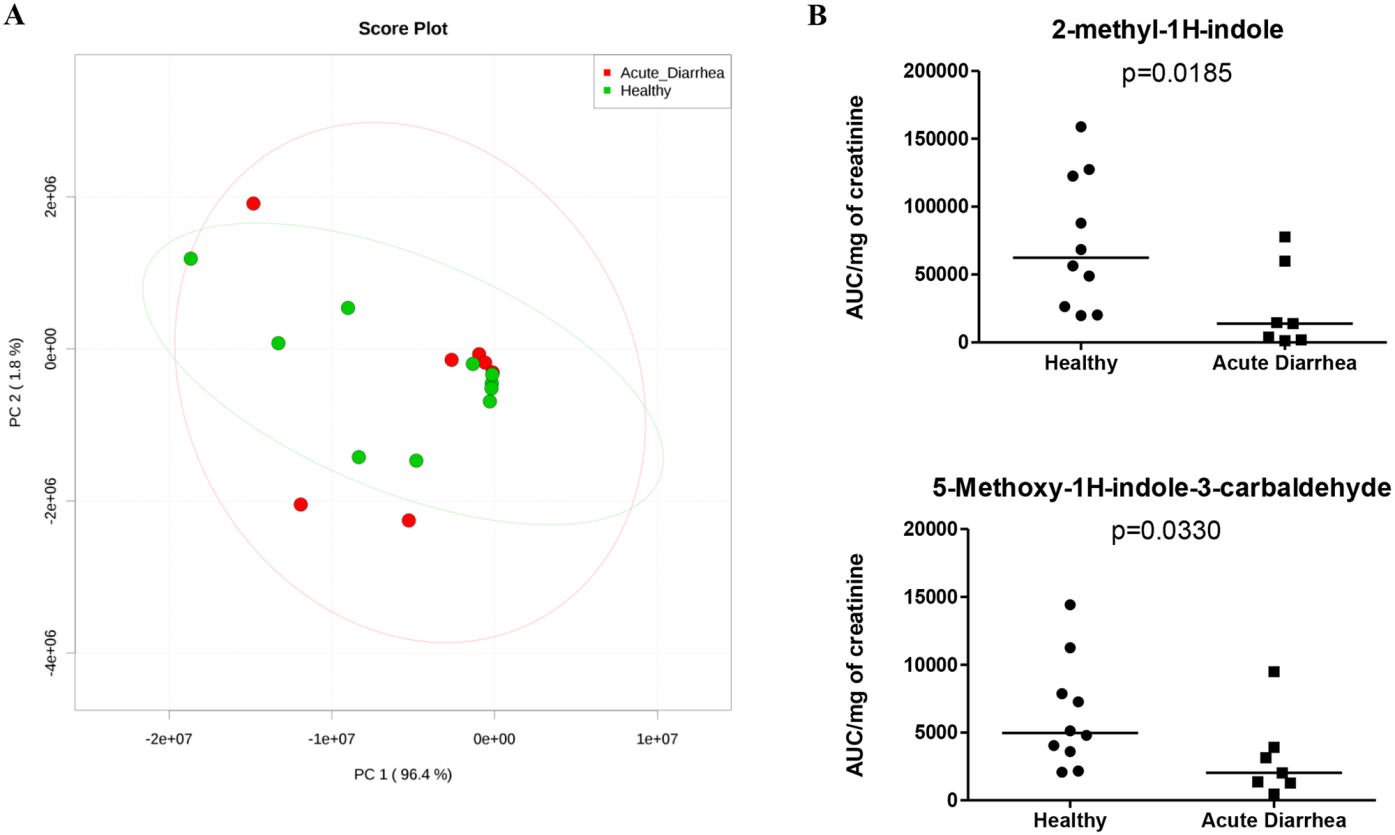
Analysis of urine metabolites. A) Principle component analysis plot where red dots represent dogs with acute diarrhea while green dots represent healthy dogs. Ellipses show the 95% confidence distribution for each group. B) Relative concentration of 2-methylindole and 5-methoxy-1H-indole-3-carbaldehyde in serum (p = 0.0185 and 0.0330, respectively).

PCA plots showed no clustering in serum or urine metabolite profiles (Figs 9 and 10, respectively) between healthy dogs and dogs with acute diarrhea. A total of 82 unique compounds were identified in serum, while 362 unique compounds were identified in urine. Of these compounds, none were significantly changed after adjusting for multiple comparisons in either of the analyzed sample types. However, in serum, unadjusted p-values showed that the concentration of kynurenic acid in serum was significantly decreased in dogs with acute diarrhea compared to healthy dogs (p = 0.0048). In addition to this comparison, the ratio between kynurenic acid (K) and tryptophan (T) concentration in the serum of healthy dogs and dogs with acute diarrhea was compared. The ratio of K/T was significantly decreased in dogs with acute diarrhea (p = 0.0036).

In urine, unadjusted p-values showed that the concentrations of 2-methyl-1H-indole and 5-Methoxy-1H-indole-3-carbaldehyde were significantly decreased in dogs with acute diarrhea compared to healthy dogs (p = 0.0185 and 0.0330, respectively).

A targeted approach was used to confirm the identity and validity of compounds found to be significantly altered from the untargeted metabolomics approach. The targeted approach for serum kynurenic acid yielded a strong correlation to the untargeted approach (ρ = 0.7213 and p = 0.0001) (S2 Fig). The targeted approach for urine 2-methyl-1H-indole and 5-Methoxy-1H-indole-3-carbaldehyde yielded a moderate to strong significant correlation to the untargeted approach (ρ = 0.6569 and p = 0.0042, ρ = 0.8162 and p<0.0001; respectively) (S3 Fig)

## Discussion

In this study we compared the fecal microbiome as well as serum and urine metabolomic profiles between healthy dogs and dogs with acute diarrhea. Our results revealed significant changes in microbial communities in dogs with AD. Consistent with our previous findings in dogs with acute diarrhea and similar to data observed in humans and mice, alpha diversity measures were significantly decreased [14, 33, 34]. Principal coordinates analysis (PCoA) plots revealed significant differences in microbiome composition between healthy dogs and dogs with AD. We identified specific bacterial groups that were altered in disease. A significant increase in *Clostridium perfringens* in dogs with AD was observed as reported previously [5, 14]. In contrast, bacterial groups decreased in AD were Bacteroidetes, *Faecalibacterium*, and an unclassified genus within Ruminococcaceae. Some of these bacterial groups are believed to be important producers of various metabolites including SCFAs [35], and consequently, we observed significant differences in SCFAs patterns between healthy and diseased dogs, with propionic acid being significantly decreased in dogs with AD. However, unexpectedly butyric acid was significantly increased in dogs with AD. Studies in humans have demonstrated the importance of SCFAs such as butyrate and propionate. For example, propionic acid has recently garnered attention for its protective effects against carcinogenesis and colorectal cancer in humans [36]. Butyrate protects against colitis by inducing apoptosis in cells with DNA damage, and also increases the expression of tight junction proteins thereby reinforcing colonic defense barriers [37–39]. Based on a decrease in *Faecalibacterium*, which has been identified as a butyrate producing bacteria in humans [40, 41], a decrease in fecal butyrate concentrations was expected in this study. This, however, was not observed. It is possible that dogs with acute diarrhea experienced decreased utilization of butyric acid by the colonic epithelium and consequently butyric acid was excreted and found in higher concentrations in the feces. *Faecalibacterium* and an unclassified genus within Ruminococcaceae shared a positive correlation with propionic acid. Alterations in fecal propionic acid could possibly be due to decreased production and/or increased absorption into the gut epithelium during stages of acute diarrhea. Therefore, it appears that the dysbiosis in AD had direct impact on concentration of SCFA and this warrants further research into potential therapeutic applications. Despite these findings, it is important to note that measuring fecal SCFAs may not directly represent colonic conditions and further studies are needed to investigate SCFA concentrations *in vivo*.

PICRUSt was used to infer putative metagenomes from16S rRNA gene profiles [16]. In general, there was a homogenous distribution of genes across broad functional categories, which has been previously noted in literature [42]. For instance, the abundance of genes belonging to metabolism (50%), Genetic Information Processing (20%), Environmental Information Processing (15%), and Cellular Processes (1–2%) were similar across healthy and diseased dogs. This similarity between both groups could be due to convergence in gene content among bacteria that inhabit the GIT. LEfSe was used to identify differentially abundant functional genes (i.e., KEGG orthologs) that had not yet been grouped into families and some notable differences were identified. The methyl-accepting chemotaxis (MAC) protein gene (K03406) was significantly increased in AD. The MAC protein is an integral component of ribose and galactose chemoreceptors in *Escherichia coli* and has been suggested to play a role in the pathogenesis of *E*. *coli* [43]. It may be hypothesized that an increase in the number of genes encoding the MAC protein may contribute to the survival of pathogenic microorganisms that ultimately cause acute diarrhea [44, 45]. A gene encoding transposases (K07483) was also found to be significantly more abundant in AD. Transposases can induce various types of genome rearrangements and are a major cause of mutations [46]. In part, they can be detrimental by inactivating housekeeping genes or impairing the chromosomes integrity [47]. Adversely, transposases are essential to horizontal gene transfer (HGT), which in turn is largely responsible for the diversity, strong selection, and co-evolution of microbiota in humans [48]. Transposases were recently identified as enzymes largely effected by HGT in a large-scale comparative metagenomic analysis of human fecal samples. Evaluating transposase and HGT activity may help determine functional factors that that contribute to GI disease. Alterations in functional genes greatly increase our understanding of the disease processes during acute diarrhea. However, given the limitations of PICRUSt as solely a predictor of metagenomic function, a true metagenomics approach is warranted and may yield more in-depth resolution.

Serum and urine metabolites were evaluated using an untargeted metabolomics approach. While we observed major changes in microbial communities, only a few serum or urine metabolites were significantly altered before adjusting for multiple comparisons. However, after adjustment this significance was lost. A possibility is that acute onset of diarrhea leads to only minor systemic changes detectable in serum or urine due to the short time of disease. This may be in contrast to chronic GI inflammation, where alterations of serum metabolites associated with oxidative stress have been observed in dogs with IBD [20]. Another explanation is possibly due to a limited sample size in conjunction with a large set of comparisons which were used for statistical adjustment for multiple comparisons. This adjustment is inherently conservative given the breadth of metabolites discovered by an untargeted metabolomics approach. Therefore, targeted assays with standards were developed to validate the serum and urine metabolites that were significantly altered based on unadjusted p-values. The results of the targeted assays correlated significantly with the untargeted metabolomics data verifying the results.

Although it has long been recognized that the enteric community of bacteria broadly impacts host health, the biochemical products they produce are only recently becoming better understood. In this study, derivatives and metabolic end products of tryptophan (i.e., 2-methylindole, 5-methoxy-1H-indole-3-carbaldehyde) were significantly decreased in the urine of dogs with acute diarrhea. These alterations may reflect a difference in uptake or utilization. While the relationship between indole and indole-like compounds is unclear in the context of dogs with acute diarrhea, it is interesting to note that indole has been shown to increase expression of genes involved in strengthening of the mucosal barrier and mucin production [49]. Also, the same study showed that indole decreased TNF-α mediated activation of NF-κB, as well as increased expression of the anti-inflammatory cytokine IL-10 [49]. Kynurenic acid, a catabolic derivative of tryptophan, was significantly decreased in the serum of dogs with AD [49]. The importance of kynurenic acid in GI disease is not yet fully understood but warrants further attention. The intestinal mucosa can monitor microbial ligands via pattern recognition receptors and microbial metabolites via G-protein coupled receptors (GPRs), and GPR35 is thought to be activated by kynurenic acid [50–52]. Recently, a study reported decreased mucosal but increased systemic levels of kynurenic acid in patients with irritable bowel syndrome (IBS) [53]. Indoleamine 2,3-dioxygenase (IDO) and tryptophan 2,3,-dioxygenase (TDO) are two enzymes implicated in catabolism of tryptophan and production of kynurenine. These enzymes play a central role in the physiological regulation of tryptophan alterations in the human body and catalyze the first and rate limiting step of tryptophan degradation along the kynurenine pathway [54]. Studies in humans with other forms of GI disease such as Crohn’s disease (CD) have suggested that aberrant handling of luminal bacteria by the innate immune system results in an activated inflammatory cascade and secretion of cytokines (i.e., TNF-α and IFN-γ) [55, 56]. These cytokines are thought to be responsible for triggering enzymatic activation of IDO and subsequent catabolism of tryptophan to form kynurenine. It may be hypothesized that the enzymatic activation of IDO could negatively impact the immune system; however this conjecture warrants further research.

The kynurenine/tryptophan ratio is used as a surrogate marker for IDO1 (i.e., the gene responsible for IDO) activity in patients with CD [57]. In a well-characterized cohort of CD patients and controls, one study found that serum tryptophan was decreased while the kynurenine/tryptophan ratio was increased in active CD [58]. While kynurenine was not measured in this study, the ratio of serum kynurenic acid to tryptophan was investigated and found to be significantly decreased in dogs with acute diarrhea. It’s not surprising that these results do not align as the kynurenine/tryptophan ratio is often used to measure up- or down-regulation of the kynurenine pathway and unfortunately in this case we were unable to readily measure kynurenine, only one of its derivatives. It may be useful to target and monitor changes in kynurenine and tryptophan in a longitudinal study which may provide a clearer picture of the role these metabolites play in disease processes.

This study had limitations. Only a small number of animals were enrolled in the disease and healthy group. In this respect, we acknowledge that results should be viewed as descriptive and are useful in justifying larger scale studies to further confirm our findings. Evaluating pet dogs living in various home environments is inherently difficult due to differences in size, weight, obesity status and sex (intact vs. not-intact). In this study, the potential influence of gender on microbial communities (S1 Fig) was investigated using the unweighted Unifrac distance matrix on all dogs and there were no significant differences between males or females (ANOSIM: p = 0.6400) regardless of sexual status (ANOSIM: p = 0.7900). Studies in humans and mice have indicated that obesity plays a role in microbiome composition [59, 60]. The current data in dogs regarding the intestinal microbiome in dogs is conflicting, and a previous study has not shown major differences between lean and obese pet dogs [61]. The body condition score (BCS, on a scale of 1–9 with 4–5 being ideal) used to differentiate between lean and obese dogs (S1 Table) was fairly heterogeneous amongst all dogs and clustering analysis of unweighted Unifrac distances revealed no significant clustering by BCS. Additionally, in some instances, only a limited amount of sample was available; therefore some samples could not be included for all analyses. Therefore, it is possible that some alterations may have been overlooked. All dogs were housed in different home environments before presentation, which may have a minor effect on GI microbiota. Diet has also been shown to alter the intestinal microbiota [62]. However, in this study, dogs in both groups were on a maintenance diet, so this fact likely had a minimal effect on microbial groups.

## Conclusions

Results of this study revealed a bacterial dysbiosis in fecal samples of dogs with acute diarrhea. Microbial diversity was significantly decreased and microbial communities differed significantly in dogs with AD. Predicted gene families were conserved across both groups of dogs. Furthermore, propionic acid was significantly correlated with a loss in SCFA producing bacteria (i.e., *Faecalibacterium*). The fecal dysbiosis was associated with significant changes in profiles of fecal SCFA and serum and urine metabolites. This suggests that acute episodes of diarrhea, even when uncomplicated, have an impact on the overall metabolic profile of the host.

## Supporting Information

S1 Fig Principal Coordinate Analysis (PCoA) of unweighted UniFrac distances of 16S rRNA genes representing clustering of samples categorized by sex (A) and sexual status (B). (PDF)

S2 Fig Comparison between serum untargeted and targeted metabolomics approach for kynurenic acid. A) Untargeted kynurenic acid. B) Targeted kynurenic acid. C) Correlation between both approaches.
(TIF)

S3 Fig Comparison between urine untargeted and targeted metabolomics approach for 2-methyl-1H-indole and 5-methoxy-1H-indole-3-carbaldehyde. A-C) Represent untargeted, targeted, and correlation results for 2-methyl-1H-indole, respectively. D-F) Represent untargeted, targeted, and correlation results for 5-methoxy-1H-indole-3-carbaldehyde, respectively.
(TIF)

S1 Table Signalment of dogs enrolled into this study. (PDF)

S2 Table Percentages of KEGG orthologs that belong to gene families at levels 1, 2, and 3. (PDF)

S3 Table Total concentrations of SCFAs and BCFAs. (PDF)

S4 Table Oligonucleotide primers/probe used for this study. (PDF)
