# Supplementary material for: Characterization of Microbial Dysbiosis and Metabolomic Changes in Dogs with Acute Diarrhea
Source: PLoS One. 2015 May 22;10(5):e0127259. doi: 10.1371/journal.pone.0127259 (PMC4441376; doi:10.1371/journal.pone.0127259)
Supplement: S1 Fig — (PDF) [file pone.0127259.s001.pdf]

A

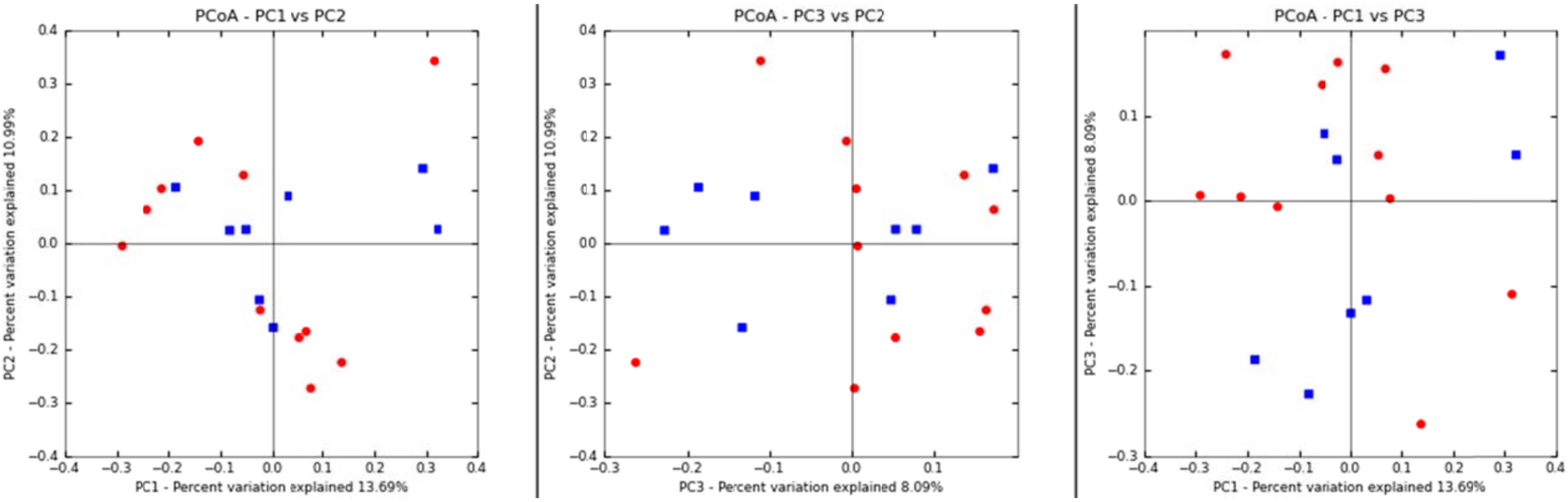

Panel A) Red circles represent female dogs and blue squares represent male dogs, both regardless of status (i.e., spayed or neutered). There was no significant difference in microbial communities between male or female when observing all dogs in the study (ANOSIM: p-value = 0.640).

B

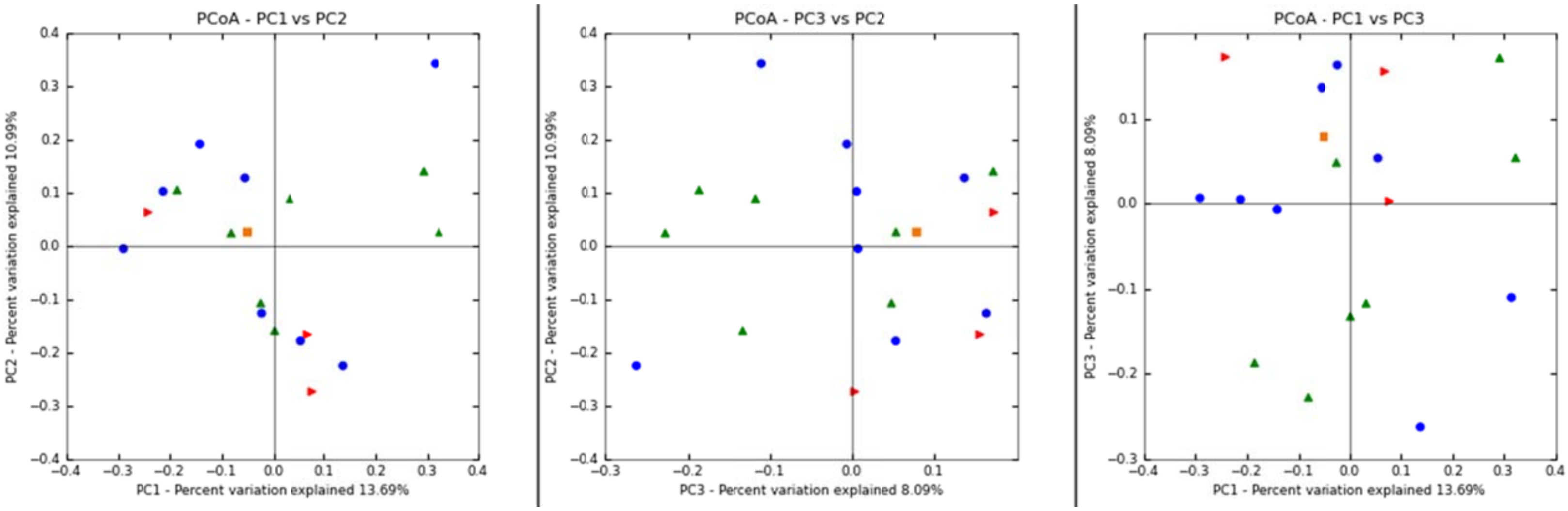

Panel B) Blue circles represent spayed females, red triangles represent intact females, green triangles represent neutered males, and yellow squares represent intact males. There was no significant difference in microbial communities between any of these groups when observing all dogs in the study (ANOSIM: p-value = 0.790).
