# Supplementary figures and images for: Characterization of Microbial Dysbiosis and Metabolomic Changes in Dogs with Acute Diarrhea
Source: PLoS One. 2015 May 22;10(5):e0127259. doi: 10.1371/journal.pone.0127259 (PMC4441376; doi:10.1371/journal.pone.0127259)

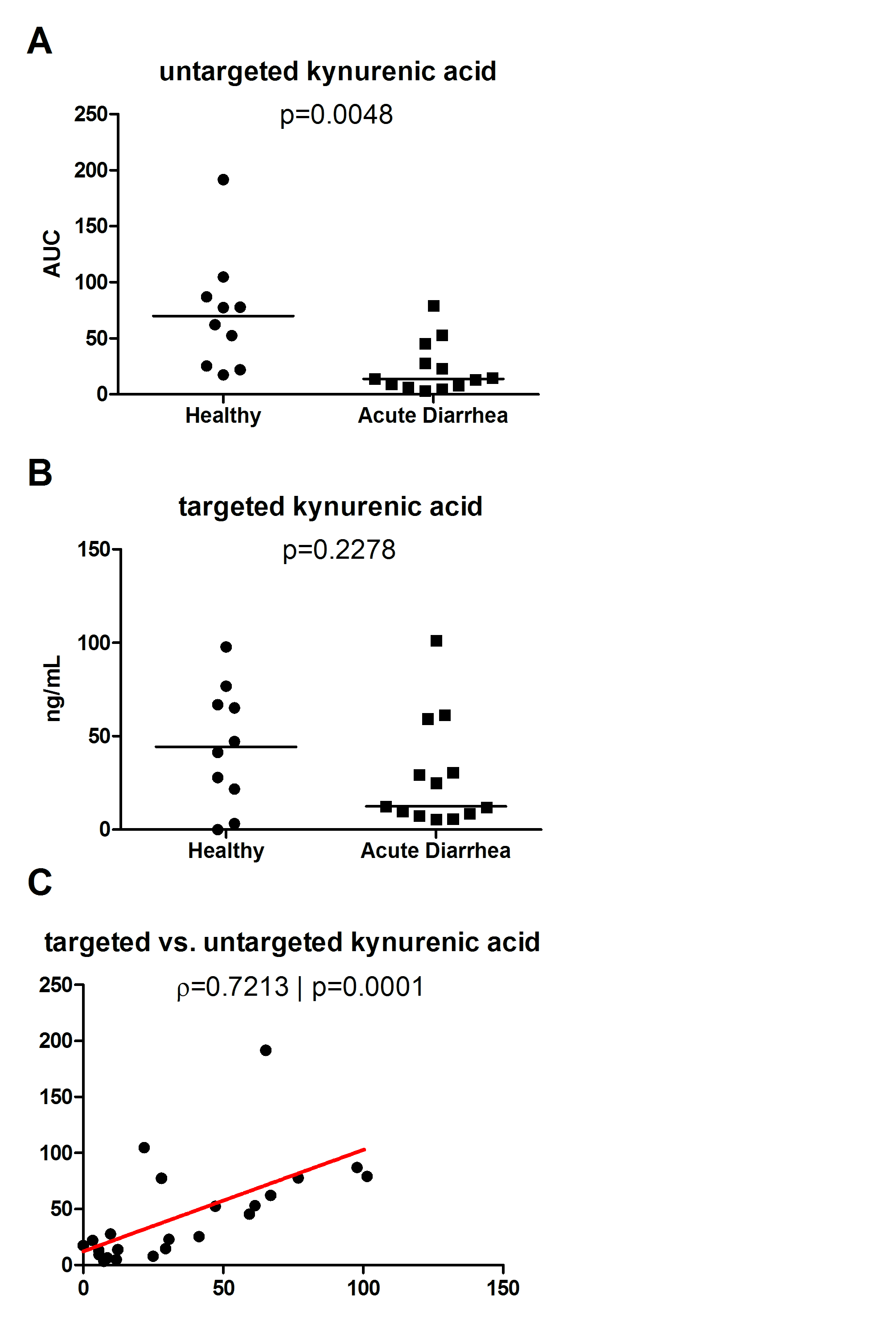

Supplement: S2 Fig — A) Untargeted kynurenic acid. B) Targeted kynurenic acid. C) Correlation between both approaches. (TIF) [file pone.0127259.s002.tif]

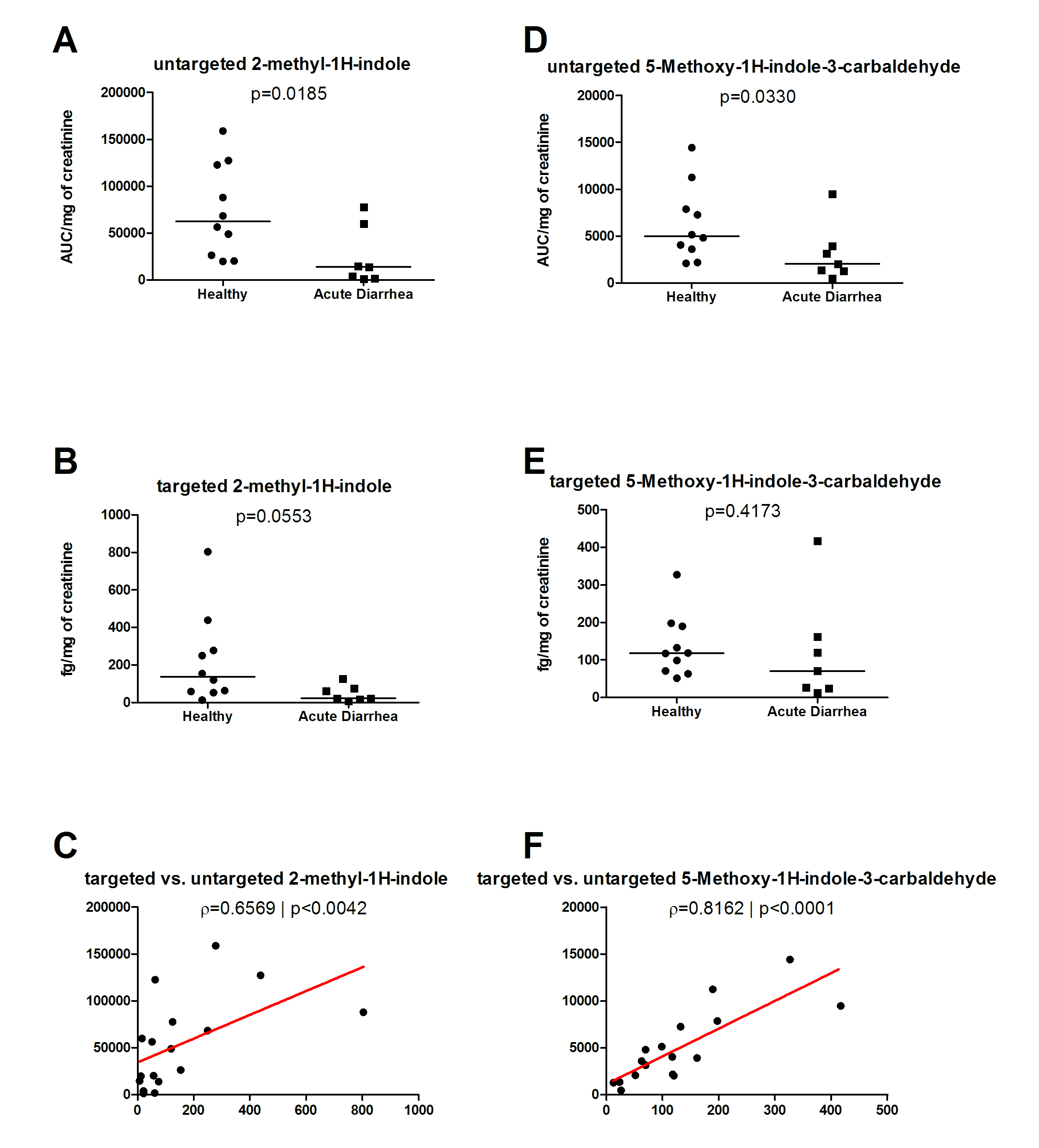

Supplement: S3 Fig — A-C) Represent untargeted, targeted, and correlation results for 2-methyl-1H-indole, respectively. D-F) Represent untargeted, targeted, and correlation results for 5-methoxy-1H-indole-3-carbaldehyde, respectively. (TIF) [file pone.0127259.s003.tif]
