# Supplementary material for: Characterization of Microbial Dysbiosis and Metabolomic Changes in Dogs with Acute Diarrhea
Source: PLoS One. 2015 May 22;10(5):e0127259. doi: 10.1371/journal.pone.0127259 (PMC4441376; doi:10.1371/journal.pone.0127259)
Supplement: S1 Table — (PDF) [file pone.0127259.s004.pdf]

Table S1: Signalment of dogs enrolled into this study.

Animal ID	Age (years)	Breed	Body Condition Score	Sex and sexual status
H1	3	Labrador	7	MN
H2	7	Boston terrier mix	7	MN
H3	9	Labrador	5	FS
H4	5	German shepherd	5	FS
H5	3	Australian Kelpie	4	F
H6	1	Labrador mix	4	F
H7	10	Weimaraner	Unknown	MN
H8	8	Miniature schnauzer	Unknown	FS
H9	12	Miniature schnauzer	Unknown	FS
H10	5	Boxer mix	5	MN
H11	3	Australian shepherd	5	FS
H12	1	Maltipoo	Unknown	FS
H13	7	Boston terrier	unknown	MN
NHD1	1	Husky/shepherd mix	5	FS
NHD2	1	Miniature pinscher	5	F
NHD3	12	American collie	6	FS
NHD4	1	Brittany spaniel	4	M
NHD5	6	Basset hound	7	FS
NHD6	1	Old English Bulldog	6	MN
AHD1	7	Labrador	4	MN
AHD2	1	Brittany spaniel	Unknown	MN
AHD3	5	Bulldog	6	MN
AHD4	3	Shetland sheepdog	5	FS
AHD5	2	Dachshund	Unknown	F
AHD6	10	American Pit Bull terrier	6	MN
AHD7	4	Pug/Beagle mix	6	FS

H = healthy; NHD = acute non-hemorrhagic diarrhea; AHD = acute hemorrhagic diarrhea

M = intact male; MN = male neutered; F = intact feamle; FS = female spayed
