# Supplementary material for: Characterization of Microbial Dysbiosis and Metabolomic Changes in Dogs with Acute Diarrhea
Source: PLoS One. 2015 May 22;10(5):e0127259. doi: 10.1371/journal.pone.0127259 (PMC4441376; doi:10.1371/journal.pone.0127259)
Supplement: S2 Table — (PDF) [file pone.0127259.s005.pdf]

**Table S2: Percentages of KEGG orthologs that belong to gene families at levels 1, 2, and 3.**

| | Median (min-max) *in percent | | |
|---|---|---|---|
| **Functional gene categories** | **Healthy** | **Acute Diarrhea** | **p-value** |
| **Level 1** | | | |
| Cellular Processes | 1.7(1.4-1.8) | 1.5(1.3-3.6) | 0.9112 |
| Environmental Information Processing | 15.3(14.7-16.4) | 15.1(12.5-17.1) | 0.9559 |
| Genetic Information Processing | 19.7(19.1-20.4) | 19.8(19.0-22.1) | 0.7718 |
| Human Diseases | 0.6(0.6-0.7) | 0.7(0.4-0.8) | 0.7452 |
| Metabolism | 49.3(47.5-49.5) | 48.7(46.1-49.5) | 0.9672 |
| None | 0.1(0.0-0.2) | 0.1(0.0-0.2) | 1.0000 |
| Organismal Systems | 0.6(0.5-0.9) | 0.6(0.5-0.8) | 0.9339 |
| Unclassified | 13.2(12.8-13.6) | 13.5(12.7-14.0) | 0.9224 |
| **Level 2** | | | |
| Cellular Processes \| Cell Growth and Death | 0.5(0.4-0.9) | 0.5(0.5-0.7) | 0.9659 |
| Cellular Processes \| Cell Motility | 0.8(0.6-1.2) | 0.9(0.7-2.4) | 0.8924 |
| Cellular Processes \| Transport and Catabolism | 0.2(0.0-0.2) | 0.1(0.1-0.2) | 0.9229 |
| Environmental Information Processing \| Membrane Transport | 14.1(13.6-15.0) | 13.7(10.9-16.2) | 0.9041 |
| Environmental Information Processing \| Signal Transduction | 1.3(0.9-1.6) | 1.2(1.0-1.6) | 0.9694 |
| Environmental Information Processing \| Signaling Molecules and Interaction | 0.1(0.1-0.2) | 0.1(0.0-0.2) | 0.9114 |
| Genetic Information Processing \| Folding, Sorting and Degradation | 2.3(2.1-2.6) | 2.4(2.2-2.7) | 1.0000 |
| Genetic Information Processing \| Replication and Repair | 9.0(7.9-9.5) | 9.0(8.5-10.0) | 0.9158 |
| Genetic Information Processing \| Transcription | 3.1(2.8-3.3) | 3.0(2.7-3.3) | 0.9874 |
| Genetic Information Processing \| Translation | 5.9(5.0-6.3) | 5.7(5.0-6.2) | 0.8403 |
| Human Diseases \| Cancers | 0.0(0.0-0.2) | 0.1(0.0-0.1) | 0.8960 |
| Human Diseases \| Immune System Diseases | 0.0(0.0-0.1) | 0.1(0.0-0.1) | 0.8633 |
| Human Diseases \| Infectious Diseases | 0.3(0.2-0.4) | 0.3(0.2-0.5) | 0.9429 |
| Human Diseases \| Metabolic Diseases | 0.1(0.0-0.2) | 0.1(0.0-0.2) | 0.9756 |
| Human Diseases \| Neurodegenerative Diseases | 0.0(0.0-0.1) | 0.0(0.0-0.1) | 1.0000 |
| Metabolism \| Amino Acid Metabolism | 10.0(9.2-10.8) | 10.6(9.2-11.8) | 1.0000 |
| Metabolism \| Biosynthesis of Other Secondary Metabolites | 1.0(0.8-1.4) | 1.0(0.8-1.3) | 1.0000 |
| Metabolism \| Carbohydrate Metabolism | 11.6(11.1-13.0) | 11.3(10.1-12.7) | 1.0000 |

| | | | |
|---|---|---|---|
| Metabolism \| Energy Metabolism | 6.3(5.4-6.8) | 5.8(5.4-6.6) | 1.0000 |
| Metabolism \| Enzyme Families | 2.1(1.8-2.3) | 2.0(1.7-2.4) | 0.8880 |
| Metabolism \| Glycan Biosynthesis and Metabolism | 1.4(1.1-1.7) | 1.3(1.1-1.9) | 0.9670 |
| Metabolism \| Lipid Metabolism | 2.6(2.3-2.9) | 2.6(2.1-3.2) | 0.9521 |
| Metabolism \| Metabolism of Cofactors and Vitamins | 4.5(3.6-4.7) | 4.3(4.0-4.8) | 0.9600 |
| Metabolism \| Metabolism of Other Amino Acids | 1.4(1.1-1.6) | 1.3(1.0-1.4) | 1.0000 |
| Metabolism \| Metabolism of Terpenoids and Polyketides | 1.6(1.3-1.8) | 1.6(1.1-1.8) | 0.9391 |
| Metabolism \| Nucleotide Metabolism | 3.9(3.4-4.6) | 3.9(3.5-4.7) | 0.8899 |
| Metabolism \| Xenobiotics Biodegradation and Metabolism | 2.1(1.6-2.3) | 2.3(1.8-2.6) | 1.0000 |
| Organismal Systems \| Digestive System | 0.0(0.0-0.0) | 0.0(0.0-0.0) | 0.8897 |
| Organismal Systems \| Endocrine System | 0.2(0.2-0.3) | 0.2(0.1-0.4) | 0.9532 |
| Organismal Systems \| Environmental Adaptation | 0.2(0.1-0.3) | 0.2(0.1-0.2) | 0.9722 |
| Organismal Systems \| Excretory System | 0.0(0.0-0.0) | 0.0(0.0-0.1) | 0.8999 |
| Organismal Systems \| Immune System | 0.1(0.0-0.2) | 0.1(0.1-0.2) | 1.0000 |
| Organismal Systems \| Nervous System | 0.1(0.1-0.1) | 0.1(0.0-0.2) | 0.8637 |
| Unclassified \| Cellular Processes and Signaling | 3.9(3.5-4.4) | 4.0(3.6-4.6) | 0.8395 |
| Unclassified \| Genetic Information Processing | 2.4(2.1-3.0) | 2.3(2.2-3.1) | 0.9808 |
| Unclassified \| Metabolism | 2.9(2.3-3.2) | 2.7(2.2-3.0) | 0.8914 |
| Unclassified \| Poorly Characterized | 4.2(3.9-4.8) | 4.3(4.0-4.9) | 0.9386 |
| **Level 3** | | | |
| Cellular Processes \| Cell Growth and Death \| Cell cycle - Caulobacter | 0.6(0.3-0.9) | 0.5(0.4-0.8) | 0.9767 |
| Cellular Processes \| Cell Motility \| Bacterial chemotaxis | 0.3(0.2-0.4) | 0.2(0.1-0.6) | 1.0000 |
| Cellular Processes \| Cell Motility \| Bacterial motility proteins | 0.2(0.0-0.2) | 0.2(0.0-1.0) | 1.0000 |
| Cellular Processes \| Cell Motility \| Cytoskeleton proteins | 0.4(0.2-0.5) | 0.4(0.3-0.6) | 0.9815 |
| Cellular Processes \| Cell Motility \| Flagellar assembly | 0.1(0.1-0.2) | 0.1(0.0-0.2) | 1.0000 |
| Cellular Processes \| Transport and Catabolism \| Lysosome | 4.2(3.4-4.4) | 4.3(2.6-4.9) | 1.0000 |
| Cellular Processes \| Transport and Catabolism \| Peroxisome | 0.5(0.4-0.6) | 0.4(0.2-0.7) | 1.0000 |
| Environmental Information Processing \| Membrane Transport \| ABC transporters | 0.4(0.2-2.3) | 0.4(0.2-1.3) | 0.9768 |
| Environmental Information Processing \| Membrane Transport \| Bacterial secretion system | 0.8(0.7-1.0) | 0.9(0.8-1.1) | 0.9647 |
| Environmental Information Processing \| Membrane Transport \| Phosphotransferase system (PTS) | 7.9(7.4-8.7) | 8.1(6.0-8.9) | 1.0000 |
| Environmental Information Processing \| Membrane Transport \| Secretion system | 0.0(0.0-0.1) | 0.0(0.0-0.1) | 1.0000 |
| Environmental Information Processing \| Membrane Transport \| Transporters | 0.1(0.0-0.2) | 0.1(0.0-0.1) | 1.0000 |
| Environmental Information Processing \| Signal Transduction \| MAPK signaling pathway - yeast | 1.1(0.7-1.4) | 1.1(0.8-1.5) | 1.0000 |
| Environmental Information Processing \| Signal Transduction \| Phosphatidylinositol signaling system | 0.1(0.1-0.2) | 0.1(0.1-0.2) | 1.0000 |
| Environmental Information Processing \| Signal Transduction \| Two-component system | 0.9(0.6-1.1) | 0.9(0.7-1.2) | 1.0000 |

| | | | |
|---|---|---|---|
| Environmental Information Processing \| Signaling Molecules and Interaction \| Bacterial toxins | 0.0(0.0-0.1) | 0.0(0.0-0.1) | 1.0000 |
| Environmental Information Processing \| Signaling Molecules and Interaction \| Cellular antigens | 0.5(0.4-0.7) | 0.5(0.3-0.7) | 0.9894 |
| Environmental Information Processing \| Signaling Molecules and Interaction \| Ion channels | 0.1(0.0-0.1) | 0.0(0.0-0.1) | 0.9715 |
| Genetic Information Processing \| Folding, Sorting and Degradation \| Chaperones and folding catalysts | 0.4(0.2-0.5) | 0.5(0.2-0.7) | 0.9839 |
| Genetic Information Processing \| Folding, Sorting and Degradation \| Proteasome | 0.2(0.2-0.4) | 0.3(0.3-0.5) | 0.9914 |
| Genetic Information Processing \| Folding, Sorting and Degradation \| Protein export | 0.5(0.3-0.6) | 0.5(0.4-0.6) | 1.0000 |
| Genetic Information Processing \| Folding, Sorting and Degradation \| Protein processing in endoplasmic reticulum | 1.5(1.4-1.7) | 1.5(1.3-1.9) | 1.0000 |
| Genetic Information Processing \| Folding, Sorting and Degradation \| RNA degradation | 3.0(2.7-3.3) | 2.8(2.6-3.4) | 1.0000 |
| Genetic Information Processing \| Folding, Sorting and Degradation \| Sulfur relay system | 0.7(0.5-0.8) | 0.7(0.5-0.8) | 0.8572 |
| Genetic Information Processing \| Replication and Repair \| Base excision repair | 1.2(0.9-1.5) | 1.3(1.0-1.7) | 1.0000 |
| Genetic Information Processing \| Replication and Repair \| Chromosome | 1.0(0.7-1.2) | 1.0(0.7-1.2) | 1.0000 |
| Genetic Information Processing \| Replication and Repair \| DNA repair and recombination proteins | 0.8(0.7-1.0) | 0.8(0.7-1.0) | 1.0000 |
| Genetic Information Processing \| Replication and Repair \| DNA replication | 0.4(0.2-0.5) | 0.4(0.2-0.5) | 1.0000 |
| Genetic Information Processing \| Replication and Repair \| DNA replication proteins | 0.2(0.1-0.2) | 0.2(0.0-0.2) | 0.9929 |
| Genetic Information Processing \| Replication and Repair \| Homologous recombination | 2.1(1.4-2.3) | 2.1(1.7-2.3) | 1.0000 |
| Genetic Information Processing \| Replication and Repair \| Mismatch repair | 1.0(0.8-1.2) | 1.0(0.9-1.1) | 1.0000 |
| Genetic Information Processing \| Replication and Repair \| Non-homologous end-joining | 1.2(1.0-1.5) | 1.1(0.8-1.6) | 1.0000 |
| Genetic Information Processing \| Replication and Repair \| Nucleotide excision repair | 0.2(0.1-0.2) | 0.2(0.0-0.2) | 1.0000 |
| Genetic Information Processing \| Transcription \| Basal transcription factors | 2.2(2.0-2.6) | 2.3(1.7-2.6) | 1.0000 |
| Genetic Information Processing \| Transcription \| RNA polymerase | 1.4(1.1-1.7) | 1.3(1.0-1.6) | 0.9686 |
| Genetic Information Processing \| Transcription \| Transcription factors | 0.0(0.0-0.1) | 0.0(0.0-0.1) | 1.0000 |
| Genetic Information Processing \| Transcription \| Transcription machinery | 0.6(0.5-0.7) | 0.5(0.4-0.9) | 1.0000 |
| Genetic Information Processing \| Translation \| Aminoacyl-tRNA biosynthesis | 0.0(0.0-0.1) | 0.0(0.0-0.1) | 1.0000 |
| Genetic Information Processing \| Translation \| Ribosome | 0.0(0.0-0.1) | 0.0(0.0-0.1) | 1.0000 |
| Genetic Information Processing \| Translation \| Ribosome Biogenesis | 0.0(0.0-0.1) | 0.0(0.0-0.1) | 0.9752 |
| Genetic Information Processing \| Translation \| Ribosome biogenesis in eukaryotes | 0.1(0.0-0.2) | 0.1(0.0-0.2) | 1.0000 |
| Genetic Information Processing \| Translation \| RNA transport | 0.1(0.1-0.2) | 0.1(0.1-0.2) | 1.0000 |
| Genetic Information Processing \| Translation \| Translation factors | 0.1(0.0-0.1) | 0.1(0.0-0.2) | 1.0000 |
| Human Diseases \| Cancers \| Pathways in cancer | 0.0(0.0-0.1) | 0.0(0.0-0.1) | 1.0000 |
| Human Diseases \| Cancers \| Prostate cancer | 0.0(0.0-0.1) | 0.0(0.0-0.1) | 1.0000 |
| Human Diseases \| Immune System Diseases \| Primary immunodeficiency | 0.0(0.0-0.1) | 0.1(0.0-0.1) | 0.9922 |
| Human Diseases \| Infectious Diseases \| Epithelial cell signaling in Helicobacter pylori infection | 1.2(0.9-1.3) | 1.1(0.9-1.5) | 1.0000 |
| Human Diseases \| Infectious Diseases \| Pertussis | 1.4(1.2-1.6) | 1.5(1.3-1.6) | 1.0000 |
| Human Diseases \| Infectious Diseases \| Staphylococcus aureus infection | 1.3(1.1-1.5) | 1.3(1.2-1.5) | 1.0000 |
| Human Diseases \| Infectious Diseases \| Tuberculosis | 0.9(0.6-1.2) | 1.0(0.8-1.3) | 0.9814 |

| | | | |
|---|---|---|---|
| Human Diseases \| Infectious Diseases \| Vibrio cholerae pathogenic cycle | 0.9(0.8-1.0) | 0.9(0.7-1.1) | 1.0000 |
| Human Diseases \| Metabolic Diseases \| Type I diabetes mellitus | 0.8(0.6-1.0) | 0.7(0.5-1.0) | 1.0000 |
| Human Diseases \| Metabolic Diseases \| Type II diabetes mellitus | 0.8(0.7-1.0) | 0.9(0.7-1.1) | 0.9702 |
| Human Diseases \| Neurodegenerative Diseases \| Alzheimer's disease | 0.1(0.0-0.2) | 0.1(0.1-0.2) | 0.9802 |
| Human Diseases \| Neurodegenerative Diseases \| Huntington's disease | 0.2(0.1-0.3) | 0.2(0.1-0.3) | 1.0000 |
| Human Diseases \| Neurodegenerative Diseases \| Parkinson's disease | 0.9(0.8-1.2) | 0.9(0.7-1.2) | 1.0000 |
| Metabolism \| Amino Acid Metabolism \| Alanine, aspartate and glutamate metabolism | 0.1(0.1-0.2) | 0.1(0.0-0.2) | 1.0000 |
| Metabolism \| Amino Acid Metabolism \| Amino acid related enzymes | 0.5(0.3-0.6) | 0.6(0.3-0.7) | 1.0000 |
| Metabolism \| Amino Acid Metabolism \| Arginine and proline metabolism | 0.7(0.5-1.0) | 0.7(0.6-0.9) | 1.0000 |
| Metabolism \| Amino Acid Metabolism \| Cysteine and methionine metabolism | 0.2(0.1-0.2) | 0.2(0.1-0.2) | 0.9975 |
| Metabolism \| Amino Acid Metabolism \| Glycine, serine and threonine metabolism | 0.1(0.1-0.1) | 0.1(0.0-0.2) | 1.0000 |
| Metabolism \| Amino Acid Metabolism \| Histidine metabolism | 0.1(0.0-0.1) | 0.1(0.0-0.1) | 1.0000 |
| Metabolism \| Amino Acid Metabolism \| Lysine biosynthesis | 0.2(0.2-0.3) | 0.2(0.1-0.3) | 1.0000 |
| Metabolism \| Amino Acid Metabolism \| Lysine degradation | 0.1(0.1-0.2) | 0.1(0.0-0.2) | 1.0000 |
| Metabolism \| Amino Acid Metabolism \| Phenylalanine metabolism | 0.3(0.2-0.5) | 0.2(0.1-0.4) | 1.0000 |
| Metabolism \| Amino Acid Metabolism \| Phenylalanine, tyrosine and tryptophan biosynthesis | 0.2(0.1-0.3) | 0.2(0.0-0.3) | 1.0000 |
| Metabolism \| Amino Acid Metabolism \| Tryptophan metabolism | 0.0(0.0-0.1) | 0.0(0.0-0.1) | 0.9861 |
| Metabolism \| Amino Acid Metabolism \| Tyrosine metabolism | 1.4(0.9-2.2) | 1.3(1.0-1.7) | 1.0000 |
| Metabolism \| Amino Acid Metabolism \| Valine, leucine and isoleucine biosynthesis | 0.1(0.0-0.2) | 0.1(0.0-0.1) | 1.0000 |
| Metabolism \| Amino Acid Metabolism \| Valine, leucine and isoleucine degradation | 0.7(0.5-0.9) | 0.7(0.5-0.8) | 1.0000 |
| Metabolism \| Biosynthesis of Other Secondary Metabolites \| beta-Lactam resistance | 0.4(0.2-0.6) | 0.4(0.3-0.7) | 1.0000 |
| Metabolism \| Biosynthesis of Other Secondary Metabolites \| Butirosin and neomycin biosynthesis | 0.5(0.4-0.8) | 0.6(0.1-0.8) | 1.0000 |
| Metabolism \| Biosynthesis of Other Secondary Metabolites \| Flavone and flavonol biosynthesis | 1.3(1.0-1.8) | 1.2(1.0-1.5) | 1.0000 |
| Metabolism \| Biosynthesis of Other Secondary Metabolites \| Isoquinoline alkaloid biosynthesis | 0.7(0.6-0.9) | 0.7(0.5-0.9) | 1.0000 |
| Metabolism \| Biosynthesis of Other Secondary Metabolites \| Novobiocin biosynthesis | 1.3(1.1-1.5) | 1.3(1.1-1.6) | 1.0000 |
| Metabolism \| Biosynthesis of Other Secondary Metabolites \| Phenylpropanoid biosynthesis | 0.5(0.4-0.7) | 0.5(0.4-0.8) | 0.9982 |
| Metabolism \| Biosynthesis of Other Secondary Metabolites \| Stilbenoid, diarylheptanoid and gingerol biosynthesis | 0.1(0.0-0.2) | 0.1(0.0-0.1) | 0.9984 |
| Metabolism \| Biosynthesis of Other Secondary Metabolites \| Streptomycin biosynthesis | 0.6(0.5-0.7) | 0.6(0.2-0.7) | 1.0000 |
| Metabolism \| Biosynthesis of Other Secondary Metabolites \| Tropane, piperidine and pyridine alkaloid biosynthesis | 1.0(0.9-1.4) | 1.2(0.9-1.3) | 1.0000 |
| Metabolism \| Carbohydrate Metabolism \| Amino sugar and nucleotide sugar metabolism | 0.5(0.4-0.8) | 0.6(0.4-0.8) | 1.0000 |
| Metabolism \| Carbohydrate Metabolism \| Ascorbate and aldarate metabolism | 1.1(0.9-1.3) | 1.1(0.8-1.3) | 1.0000 |
| Metabolism \| Carbohydrate Metabolism \| Butanoate metabolism | 1.2(1.0-1.5) | 1.2(0.9-1.3) | 0.9757 |
| Metabolism \| Carbohydrate Metabolism \| C5-Branched dibasic acid metabolism | 0.8(0.5-1.0) | 0.7(0.6-0.9) | 1.0000 |
| Metabolism \| Carbohydrate Metabolism \| Citrate cycle (TCA cycle) | 0.9(0.8-1.2) | 1.0(0.7-1.1) | 1.0000 |
| Metabolism \| Carbohydrate Metabolism \| Fructose and mannose metabolism | 1.5(1.3-1.9) | 1.5(1.2-2.2) | 1.0000 |

| | | | |
|---|---|---|---|
| Metabolism \| Carbohydrate Metabolism \| Galactose metabolism | 0.6(0.5-0.9) | 0.7(0.4-0.8) | 0.9982 |
| Metabolism \| Carbohydrate Metabolism \| Glycolysis / Gluconeogenesis | 1.2(0.9-1.5) | 1.3(0.8-1.4) | 1.0000 |
| Metabolism \| Carbohydrate Metabolism \| Glyoxylate and dicarboxylate metabolism | 0.5(0.4-0.7) | 0.4(0.3-0.6) | 1.0000 |
| Metabolism \| Carbohydrate Metabolism \| Inositol phosphate metabolism | 0.4(0.2-0.6) | 0.4(0.2-0.6) | 1.0000 |
| Metabolism \| Carbohydrate Metabolism \| Pentose and glucuronate interconversions | 0.2(0.1-0.4) | 0.2(0.1-0.3) | 1.0000 |
| Metabolism \| Carbohydrate Metabolism \| Pentose phosphate pathway | 1.9(1.6-2.1) | 1.7(1.4-2.1) | 1.0000 |
| Metabolism \| Carbohydrate Metabolism \| Propanoate metabolism | 0.2(0.2-0.4) | 0.3(0.1-0.5) | 1.0000 |
| Metabolism \| Carbohydrate Metabolism \| Pyruvate metabolism | 0.0(0.0-0.1) | 0.1(0.0-0.1) | 1.0000 |
| Metabolism \| Carbohydrate Metabolism \| Starch and sucrose metabolism | 0.3(0.2-0.4) | 0.2(0.1-0.5) | 1.0000 |
| Metabolism \| Energy Metabolism \| Carbon fixation in photosynthetic organisms | 0.1(0.0-0.1) | 0.1(0.0-0.2) | 1.0000 |
| Metabolism \| Energy Metabolism \| Carbon fixation pathways in prokaryotes | 0.1(0.0-0.1) | 0.1(0.0-0.2) | 1.0000 |
| Metabolism \| Energy Metabolism \| Methane metabolism | 1.0(0.8-1.1) | 0.8(0.7-1.2) | 1.0000 |
| Metabolism \| Energy Metabolism \| Nitrogen metabolism | 0.1(0.1-0.2) | 0.1(0.0-0.2) | 1.0000 |
| Metabolism \| Energy Metabolism \| Oxidative phosphorylation | 0.5(0.3-0.7) | 0.6(0.3-0.8) | 1.0000 |
| Metabolism \| Energy Metabolism \| Photosynthesis | 0.2(0.2-0.4) | 0.2(0.1-0.3) | 1.0000 |
| Metabolism \| Energy Metabolism \| Photosynthesis proteins | 0.4(0.4-0.7) | 0.5(0.3-0.8) | 1.0000 |
| Metabolism \| Energy Metabolism \| Sulfur metabolism | 0.5(0.4-0.7) | 0.5(0.4-0.6) | 1.0000 |
| Metabolism \| Enzyme Families \| Peptidases | 0.1(0.0-0.2) | 0.1(0.0-0.2) | 0.9820 |
| Metabolism \| Enzyme Families \| Protein kinases | 0.5(0.3-0.7) | 0.5(0.3-0.6) | 1.0000 |
| Metabolism \| Glycan Biosynthesis and Metabolism \| Glycosaminoglycan degradation | 0.0(0.0-0.1) | 0.0(0.0-0.1) | 1.0000 |
| Metabolism \| Glycan Biosynthesis and Metabolism \| Glycosphingolipid biosynthesis - ganglio series | 0.0(0.0-0.1) | 0.1(0.0-0.1) | 0.9729 |
| Metabolism \| Glycan Biosynthesis and Metabolism \| Glycosphingolipid biosynthesis - globo series | 0.1(0.0-0.1) | 0.1(0.0-0.1) | 1.0000 |
| Metabolism \| Glycan Biosynthesis and Metabolism \| Glycosyltransferases | 0.1(0.1-0.3) | 0.1(0.0-0.2) | 1.0000 |
| Metabolism \| Glycan Biosynthesis and Metabolism \| Lipopolysaccharide biosynthesis | 0.4(0.3-0.6) | 0.4(0.2-0.5) | 1.0000 |
| Metabolism \| Glycan Biosynthesis and Metabolism \| Lipopolysaccharide biosynthesis proteins | 0.0(0.0-0.0) | 0.0(0.0-0.0) | 1.0000 |
| Metabolism \| Glycan Biosynthesis and Metabolism \| N-Glycan biosynthesis | 0.4(0.4-0.6) | 0.4(0.2-0.7) | 1.0000 |
| Metabolism \| Glycan Biosynthesis and Metabolism \| Other glycan degradation | 0.6(0.5-0.6) | 0.6(0.4-0.7) | 1.0000 |
| Metabolism \| Glycan Biosynthesis and Metabolism \| Peptidoglycan biosynthesis | 0.5(0.4-0.7) | 0.6(0.3-0.7) | 1.0000 |
| Metabolism \| Lipid Metabolism \| Arachidonic acid metabolism | 1.0(0.7-1.5) | 1.2(1.0-1.4) | 1.0000 |
| Metabolism \| Lipid Metabolism \| Biosynthesis of unsaturated fatty acids | 0.1(0.0-0.1) | 0.1(0.0-0.1) | 1.0000 |
| Metabolism \| Lipid Metabolism \| Ether lipid metabolism | 0.2(0.1-0.4) | 0.3(0.2-0.5) | 1.0000 |
| Metabolism \| Lipid Metabolism \| Fatty acid biosynthesis | 0.5(0.3-0.7) | 0.5(0.3-0.7) | 1.0000 |
| Metabolism \| Lipid Metabolism \| Fatty acid metabolism | 0.2(0.1-0.2) | 0.2(0.1-0.3) | 1.0000 |
| Metabolism \| Lipid Metabolism \| Glycerolipid metabolism | 0.2(0.2-0.4) | 0.2(0.1-0.4) | 0.9843 |
| Metabolism \| Lipid Metabolism \| Glycerophospholipid metabolism | 0.1(0.0-0.2) | 0.1(0.1-0.2) | 1.0000 |

| | | | |
|---|---|---|---|
| Metabolism \| Lipid Metabolism \| Linoleic acid metabolism | 0.1(0.0-0.2) | 0.1(0.1-0.3) | 1.0000 |
| Metabolism \| Lipid Metabolism \| Lipid biosynthesis proteins | 0.1(0.0-0.2) | 0.1(0.1-0.4) | 0.9710 |
| Metabolism \| Lipid Metabolism \| Primary bile acid biosynthesis | 0.0(0.0-0.1) | 0.0(0.0-0.1) | 1.0000 |
| Metabolism \| Lipid Metabolism \| Secondary bile acid biosynthesis | 0.2(0.1-0.3) | 0.2(0.2-0.4) | 1.0000 |
| Metabolism \| Lipid Metabolism \| Sphingolipid metabolism | 0.1(0.0-0.2) | 0.1(0.0-0.1) | 1.0000 |
| Metabolism \| Lipid Metabolism \| Steroid hormone biosynthesis | 0.2(0.2-0.3) | 0.2(0.1-0.5) | 0.9884 |
| Metabolism \| Lipid Metabolism \| Synthesis and degradation of ketone bodies | 0.1(0.0-0.2) | 0.1(0.1-0.2) | 1.0000 |
| Metabolism \| Metabolism of Cofactors and Vitamins \| Biotin metabolism | 0.0(0.0-0.1) | 0.0(0.0-0.1) | 1.0000 |
| Metabolism \| Metabolism of Cofactors and Vitamins \| Folate biosynthesis | 0.2(0.0-0.3) | 0.2(0.0-0.3) | 0.9897 |
| Metabolism \| Metabolism of Cofactors and Vitamins \| Lipoic acid metabolism | 0.1(0.0-0.3) | 0.2(0.1-0.3) | 1.0000 |
| Metabolism \| Metabolism of Cofactors and Vitamins \| Nicotinate and nicotinamide metabolism | 0.2(0.1-0.4) | 0.2(0.1-0.4) | 1.0000 |
| Metabolism \| Metabolism of Cofactors and Vitamins \| One carbon pool by folate | 0.5(0.4-0.7) | 0.5(0.4-0.7) | 0.9807 |
| Metabolism \| Metabolism of Cofactors and Vitamins \| Pantothenate and CoA biosynthesis | 0.2(0.1-0.2) | 0.2(0.1-0.3) | 1.0000 |
| Metabolism \| Metabolism of Cofactors and Vitamins \| Porphyrin and chlorophyll metabolism | 0.1(0.0-0.1) | 0.0(0.0-0.1) | 1.0000 |
| Metabolism \| Metabolism of Cofactors and Vitamins \| Retinol metabolism | 2.1(1.8-2.5) | 2.2(2.0-2.5) | 0.9969 |
| Metabolism \| Metabolism of Cofactors and Vitamins \| Riboflavin metabolism | 1.8(1.6-2.3) | 1.9(1.6-2.3) | 1.0000 |
| Metabolism \| Metabolism of Cofactors and Vitamins \| Thiamine metabolism | 0.2(0.0-0.2) | 0.2(0.1-0.2) | 0.9916 |
| Metabolism \| Metabolism of Cofactors and Vitamins \| Ubiquinone and other terpenoid-quinone biosynthesis | 0.2(0.1-0.4) | 0.3(0.2-0.4) | 0.9956 |
| Metabolism \| Metabolism of Cofactors and Vitamins \| Vitamin B6 metabolism | 0.1(0.0-0.2) | 0.2(0.1-0.3) | 1.0000 |
| Metabolism \| Metabolism of Other Amino Acids \| beta-Alanine metabolism | 0.4(0.2-0.6) | 0.4(0.2-0.6) | 1.0000 |
| Metabolism \| Metabolism of Other Amino Acids \| Cyanoamino acid metabolism | 0.1(0.0-0.2) | 0.1(0.0-0.2) | 1.0000 |
| Metabolism \| Metabolism of Other Amino Acids \| D-Alanine metabolism | 0.1(0.0-0.1) | 0.1(0.0-0.1) | 1.0000 |
| Metabolism \| Metabolism of Other Amino Acids \| D-Arginine and D-ornithine metabolism | 0.4(0.3-0.4) | 0.3(0.2-0.4) | 1.0000 |
| Metabolism \| Metabolism of Other Amino Acids \| D-Glutamine and D-glutamate metabolism | 0.1(0.0-0.1) | 0.0(0.0-0.1) | 1.0000 |
| Metabolism \| Metabolism of Other Amino Acids \| Glutathione metabolism | 0.1(0.0-0.2) | 0.0(0.0-0.1) | 1.0000 |
| Metabolism \| Metabolism of Other Amino Acids \| Phosphonate and phosphinate metabolism | 0.3(0.2-0.4) | 0.3(0.2-0.4) | 1.0000 |
| Metabolism \| Metabolism of Other Amino Acids \| Selenocompound metabolism | 0.1(0.0-0.2) | 0.1(0.1-0.2) | 0.9915 |
| Metabolism \| Metabolism of Other Amino Acids \| Taurine and hypotaurine metabolism | 0.1(0.0-0.2) | 0.1(0.0-0.2) | 0.9984 |
| Metabolism \| Metabolism of Terpenoids and Polyketides \| Biosynthesis of ansamycins | 0.1(0.0-0.2) | 0.1(0.0-0.1) | 1.0000 |
| Metabolism \| Metabolism of Terpenoids and Polyketides \| Biosynthesis of siderophore group nonribosomal peptides | 0.1(0.0-0.1) | 0.1(0.0-0.2) | 1.0000 |
| Metabolism \| Metabolism of Terpenoids and Polyketides \| Biosynthesis of vancomycin group antibiotics | 0.1(0.0-0.2) | 0.1(0.0-0.1) | 1.0000 |
| Metabolism \| Metabolism of Terpenoids and Polyketides \| Geraniol degradation | 0.0(0.0-0.1) | 0.1(0.0-0.1) | 1.0000 |
| Metabolism \| Metabolism of Terpenoids and Polyketides \| Limonene and pinene degradation | 0.2(0.1-0.2) | 0.2(0.1-0.4) | 1.0000 |
| Metabolism \| Metabolism of Terpenoids and Polyketides \| Polyketide sugar unit biosynthesis | 0.0(0.0-0.1) | 0.0(0.0-0.1) | 1.0000 |
| Metabolism \| Metabolism of Terpenoids and Polyketides \| Prenyltransferases | 0.1(0.0-0.1) | 0.0(0.0-0.1) | 0.9940 |

| | | | |
|---|---|---|---|
| Metabolism \| Metabolism of Terpenoids and Polyketides \| Terpenoid backbone biosynthesis | 0.0(0.0-0.1) | 0.0(0.0-0.1) | 1.0000 |
| Metabolism \| Metabolism of Terpenoids and Polyketides \| Tetracycline biosynthesis | 0.1(0.1-0.3) | 0.1(0.1-0.2) | 1.0000 |
| Metabolism \| Metabolism of Terpenoids and Polyketides \| Zeatin biosynthesis | 0.1(0.0-0.2) | 0.1(0.0-0.2) | 1.0000 |
| Metabolism \| Nucleotide Metabolism \| Purine metabolism | 0.1(0.0-0.1) | 0.1(0.0-0.2) | 0.9852 |
| Metabolism \| Nucleotide Metabolism \| Pyrimidine metabolism | 0.0(0.0-0.0) | 0.0(0.0-0.0) | 1.0000 |
| Metabolism \| Xenobiotics Biodegradation and Metabolism \| Aminobenzoate degradation | 0.2(0.1-0.2) | 0.2(0.1-0.2) | 1.0000 |
| Metabolism \| Xenobiotics Biodegradation and Metabolism \| Atrazine degradation | 0.4(0.2-0.5) | 0.3(0.1-0.5) | 1.0000 |
| Metabolism \| Xenobiotics Biodegradation and Metabolism \| Benzoate degradation | 1.0(0.9-1.3) | 1.1(0.9-1.2) | 1.0000 |
| Metabolism \| Xenobiotics Biodegradation and Metabolism \| Bisphenol degradation | 0.1(0.1-0.3) | 0.2(0.2-0.4) | 1.0000 |
| Metabolism \| Xenobiotics Biodegradation and Metabolism \| Caprolactam degradation | 0.1(0.1-0.2) | 0.2(0.1-0.3) | 1.0000 |
| Metabolism \| Xenobiotics Biodegradation and Metabolism \| Chloroalkane and chloroalkene degradation | 0.5(0.2-0.6) | 0.5(0.3-0.7) | 1.0000 |
| Metabolism \| Xenobiotics Biodegradation and Metabolism \| Chlorocyclohexane and chlorobenzene degradation | 1.2(1.0-1.5) | 1.3(1.0-1.6) | 1.0000 |
| Metabolism \| Xenobiotics Biodegradation and Metabolism \| Dioxin degradation | 0.7(0.5-0.8) | 0.6(0.4-1.0) | 1.0000 |
| Metabolism \| Xenobiotics Biodegradation and Metabolism \| Drug metabolism - cytochrome P450 | 0.8(0.5-1.1) | 0.7(0.6-1.0) | 1.0000 |
| Metabolism \| Xenobiotics Biodegradation and Metabolism \| Drug metabolism - other enzymes | 0.2(0.1-0.4) | 0.1(0.1-0.2) | 1.0000 |
| Metabolism \| Xenobiotics Biodegradation and Metabolism \| Ethylbenzene degradation | 1.0(0.7-1.1) | 0.8(0.7-1.1) | 1.0000 |
| Metabolism \| Xenobiotics Biodegradation and Metabolism \| Metabolism of xenobiotics by cytochrome P450 | 0.4(0.3-0.5) | 0.3(0.2-0.6) | 0.9868 |
| Metabolism \| Xenobiotics Biodegradation and Metabolism \| Naphthalene degradation | 0.0(0.0-0.1) | 0.0(0.0-0.1) | 1.0000 |
| Metabolism \| Xenobiotics Biodegradation and Metabolism \| Nitrotoluene degradation | 0.1(0.1-0.3) | 0.2(0.1-0.3) | 1.0000 |
| Metabolism \| Xenobiotics Biodegradation and Metabolism \| Polycyclic aromatic hydrocarbon degradation | 0.8(0.7-1.0) | 0.8(0.5-1.1) | 1.0000 |
| Metabolism \| Xenobiotics Biodegradation and Metabolism \| Styrene degradation | 0.1(0.0-0.2) | 0.1(0.0-0.1) | 1.0000 |
| Metabolism \| Xenobiotics Biodegradation and Metabolism \| Toluene degradation | 0.0(0.0-0.1) | 0.1(0.0-0.1) | 1.0000 |
| Metabolism \| Xenobiotics Biodegradation and Metabolism \| Xylene degradation | 1.3(1.1-1.6) | 1.3(1.0-1.5) | 1.0000 |
| Organismal Systems \| Digestive System \| Bile secretion | 1.1(0.8-1.2) | 1.0(0.8-1.3) | 0.9786 |
| Organismal Systems \| Digestive System \| Carbohydrate digestion and absorption | 3.3(2.7-3.8) | 3.2(3.0-3.6) | 1.0000 |
| Organismal Systems \| Digestive System \| Mineral absorption | 0.0(0.0-0.1) | 0.0(0.0-0.6) | 1.0000 |
| Organismal Systems \| Endocrine System \| Adipocytokine signaling pathway | 0.0(0.0-0.1) | 0.0(0.0-0.1) | 1.0000 |
| Organismal Systems \| Endocrine System \| Insulin signaling pathway | 0.0(0.0-0.0) | 0.0(0.0-0.0) | 1.0000 |
| Organismal Systems \| Endocrine System \| PPAR signaling pathway | 0.0(0.0-0.0) | 0.0(0.0-0.0) | 1.0000 |
| Organismal Systems \| Endocrine System \| Progesterone-mediated oocyte maturation | 0.0(0.0-0.0) | 0.0(0.0-0.0) | 1.0000 |
| Organismal Systems \| Environmental Adaptation \| Plant-pathogen interaction | 0.0(0.0-0.0) | 0.0(0.0-0.0) | 0.9939 |
| Organismal Systems \| Excretory System \| Proximal tubule bicarbonate reclamation | 0.0(0.0-0.0) | 0.0(0.0-0.0) | 1.0000 |
| Organismal Systems \| Immune System \| Antigen processing and presentation | 0.0(0.0-0.0) | 0.0(0.0-0.0) | 1.0000 |
| Organismal Systems \| Immune System \| NOD-like receptor signaling pathway | 0.0(0.0-0.0) | 0.0(0.0-0.1) | 0.9857 |
| Organismal Systems \| Immune System \| RIG-I-like receptor signaling pathway | 0.0(0.0-0.0) | 0.0(0.0-0.0) | 0.9865 |

| | | | |
|---|---|---|---|
| Organismal Systems \| Nervous System \| Glutamatergic synapse | 0.0(0.0-0.0) | 0.0(0.0-0.0) | 1.0000 |
| Unclassified \| Cellular Processes and Signaling \| Cell division | 0.0(0.0-0.0) | 0.0(0.0-0.0) | 1.0000 |
| Unclassified \| Cellular Processes and Signaling \| Cell motility and secretion | 0.0(0.0-0.0) | 0.0(0.0-0.1) | 1.0000 |
| Unclassified \| Cellular Processes and Signaling \| Electron transfer carriers | 0.0(0.0-0.0) | 0.0(0.0-0.1) | 1.0000 |
| Unclassified \| Cellular Processes and Signaling \| Germination | 0.0(0.0-0.1) | 0.0(0.0-0.1) | 1.0000 |
| Unclassified \| Cellular Processes and Signaling \| Inorganic ion transport and metabolism | 0.0(0.0-0.0) | 0.0(0.0-0.1) | 1.0000 |
| Unclassified \| Cellular Processes and Signaling \| Membrane and intracellular structural molecules | 0.0(0.0-0.0) | 0.0(0.0-0.0) | 1.0000 |
| Unclassified \| Cellular Processes and Signaling \| Other ion-coupled transporters | 0.0(0.0-0.0) | 0.0(0.0-0.0) | 1.0000 |
| Unclassified \| Cellular Processes and Signaling \| Other transporters | 0.0(0.0-0.0) | 0.0(0.0-0.0) | 1.0000 |
| Unclassified \| Cellular Processes and Signaling \| Pores ion channels | 0.0(0.0-0.0) | 0.0(0.0-0.1) | 1.0000 |
| Unclassified \| Cellular Processes and Signaling \| Signal transduction mechanisms | 0.0(0.0-0.1) | 0.0(0.0-0.2) | 1.0000 |
| Unclassified \| Cellular Processes and Signaling \| Sporulation | 0.0(0.0-0.0) | 0.0(0.0-0.0) | 1.0000 |
| Unclassified \| Genetic Information Processing \| Protein folding and associated processing | 0.0(0.0-0.0) | 0.0(0.0-0.0) | 1.0000 |
| Unclassified \| Genetic Information Processing \| Replication, recombination and repair proteins | 0.0(0.0-0.0) | 0.0(0.0-0.0) | 1.0000 |
| Unclassified \| Genetic Information Processing \| Restriction enzyme | 0.0(0.0-0.0) | 0.0(0.0-0.0) | 0.6076 |
| Unclassified \| Genetic Information Processing \| Translation proteins | 0.0(0.0-0.0) | 0.0(0.0-0.0) | 1.0000 |
| Unclassified \| Metabolism \| Amino acid metabolism | 0.0(0.0-0.0) | 0.0(0.0-0.0) | 0.9975 |
| Unclassified \| Metabolism \| Biosynthesis and biodegradation of secondary metabolites | 0.0(0.0-0.0) | 0.0(0.0-0.0) | 1.0000 |
| Unclassified \| Metabolism \| Carbohydrate metabolism | 0.0(0.0-0.1) | 0.0(0.0-0.1) | 1.0000 |
| Unclassified \| Metabolism \| Energy metabolism | 0.0(0.0-0.0) | 0.0(0.0-0.0) | 1.0000 |
| Unclassified \| Metabolism \| Glycan biosynthesis and metabolism | 0.0(0.0-0.0) | 0.0(0.0-0.0) | 1.0000 |
| Unclassified \| Metabolism \| Lipid metabolism | 0.0(0.0-0.0) | 0.0(0.0-0.0) | 0.9842 |
| Unclassified \| Metabolism \| Metabolism of cofactors and vitamins | 0.0(0.0-0.1) | 0.0(0.0-0.0) | 1.0000 |
| Unclassified \| Metabolism \| Nucleotide metabolism | 0.0(0.0-0.0) | 0.0(0.0-0.0) | 0.9711 |
| Unclassified \| Metabolism \| Others | 0.0(0.0-0.0) | 0.0(0.0-0.0) | 1.0000 |
| Unclassified \| Poorly Characterized \| Function unknown | 0.0(0.0-0.0) | 0.0(0.0-0.0) | 1.0000 |
| Unclassified \| Poorly Characterized \| General function prediction only | 0.0(0.0-0.0) | 0.0(0.0-0.0) | 1.0000 |

P-value adjusted based on the Benjamini and Hochberg false discovery rate.
