# Supplementary material for: Characterization of Microbial Dysbiosis and Metabolomic Changes in Dogs with Acute Diarrhea
Source: PLoS One. 2015 May 22;10(5):e0127259. doi: 10.1371/journal.pone.0127259 (PMC4441376; doi:10.1371/journal.pone.0127259)
Supplement: S3 Table — (PDF) [file pone.0127259.s006.pdf]

Table S3: Total concentrations of SCFAs and BCFAs.

	median (min-max) *µmol/g dry feces		
	**Healthy**	**Acute Diarrhea**	**p-value**
**acetic acid**	186.0(84.9-420.5)	238.8(83.1-700.0)	0.7285
**propionic acid**	98.6(34.2-260.9)	48.7(0.2-109.5)	0.1652
**butyric acid**	17.2(9.4-54.2)	58.2(8.7-285.1)	0.1923
**isobutyric acid**	4.3(2.0-6.8)	3.2(0.0-4.3)	0.1578
**isoveleric acid**	4.6(1.5-6.0)	3.8(0.0-6.2)	0.7717
**valeric acid**	0.4(0.1-2.1)	0.1(0.0-8.3)	0.1926

P-value adjusted based on the Benjamini and Hochberg False Discovery Rate.
