# Supplementary material for: Characterization of Microbial Dysbiosis and Metabolomic Changes in Dogs with Acute Diarrhea
Source: PLoS One. 2015 May 22;10(5):e0127259. doi: 10.1371/journal.pone.0127259 (PMC4441376; doi:10.1371/journal.pone.0127259)
Supplement: S4 Table — (PDF) [file pone.0127259.s007.pdf]

Table S4. Oligonucleotide primers/probe used for this study.

Primer Sequence (5'-3')	Target	Annealing temperature (ºC)
F-AGCAGTAGGGAATCTTCCA	*Lactobacillus*	58
R-CACCGCTACACATGGAG		
F-TCGCGTCYGGTGTGAAAG	*Bifidobacterium*	60
R-CCACATCCAGCRTCCAC		
F-GTTAATACCTTTGCTCATTGA	*Escherichia coli*	55
R-ACCAGGGTATCTAATCCTGTT		
F-CGCATAACGTTGAAAGATGG	*C. perfringens*	58
R-CCTTGGTAGGCCGTTACCC		
Probe-TCATCATTCAACCAAAGGAGCAATCC		

F = forward primer; R = reverse primer.
